# Higher dietary acid load potentially increases serum triglyceride and obesity prevalence in adults: An updated systematic review and meta-analysis

**DOI:** 10.1371/journal.pone.0216547

**Published:** 2019-05-09

**Authors:** Mahdieh Abbasalizad Farhangi, Leila Nikniaz, Zeinab Nikniaz

**Affiliations:** 1 Research Center for Evidence Based Medicine, Health Management and Safety Promotion Research Institute, Tabriz University of Medical Sciences, Tabriz, Iran; 2 Drug Applied Research Center, Tabriz University of Medical Sciences, Tabriz, Iran; 3 Tabriz Health Services Management Research Center, Health Management and Safety Promotion Research Institute, Tabriz University of Medical Sciences, Tabriz, Iran; 4 Liver and Gastrointestinal Disease Research Center, Tabriz University of Medical Sciences, Tabriz, Iran; University Magna Graecia of Catanzaro, ITALY

## Abstract

**Background:**

In the current meta-analysis, we aimed to systematically review and summarize the eligible studies evaluating the association between dietary acid load in terms of potential renal acid load (PRAL) and net-endogenous acid production (NEAP) with anthropometric parameters and serum lipids in adult population.

**Methods:**

In a systematic search from PubMed, Scopus, Web of Sciences and Cochrane electronic databases up to December 2018, relevant studies were included. Cross-sectional, case control or cohort studies evaluating the association between PRAL and NEAP with the mean values of body mass index (BMI), waist circumference (WC), low and high density lipoprotein cholesterol (LDL, HDL), triglyceride (TG), total cholesterol (TC) and the prevalence of obesity were included.

**Results:**

According to our results, having higher dietary acid load content in terms of high PRAL scores was associated with higher triglyceride concentrations (weighted mean difference (WMD): 3.468; confidence interval (CI): -0.231, 7.166, P = 0.04) and higher obesity prevalence (30% and 27% in highest versus lowest categories). Accordingly, being in the highest category of NEAP was associated with higher prevalence of obesity (25% and 22% in highest versus lowest category). In subgroup analysis, higher PRAL scores was associated with higher BMI in women (WMD: 0.122; CI: -0.001, 0.245; P = 0.049) and higher NEAP in men (WMD: 0.890; CI: 0.430, 1.350; P < 0.001). There was no association between dietary acid load and other studied parameters.

**Conclusions:**

In the current meta-analysis, high dietary acid load content was associated with higher serum triglyceride concentrations and higher obesity prevalence. Reducing dietary acid load content might be a useful preventive strategy against obesity and metabolic disorders.

## Introduction

Overweight and obesity, defined as abnormal or excessive fat accumulation in the body, is a major growing epidemic health problem; according to the world health organization report, since 1975, the worldwide obesity has nearly tripled and in 2016, more than 1.9 billion and over 650 million of adults were overweight and obese comprising 39% and 13% respectively [1]. It has been estimated that the direct costs of obesity accounted for 6.8%. (or US$ 70 billion) of total health care—related costs; moreover, huge indirect costs including losing workdays, disabilities, physician visits, and premature mortality alongside with intangible costs including reduced quality of life and impaired mental health because of weight dissatisfaction should also be addressed [2]. Increased prevalence of obesity consequently leads to increased rates of diabetes, metabolic syndrome, cardiovascular events and numerous other chronic obesity-related disease further highlights the importance of preventive strategies. Among them, modifying nutrition and diet-related behaviors must be one of the most effective strategies [3]. Recently, the role of diet- related low-level metabolic acidosis in the pathogenesis of metabolic disorders including metabolic syndrome, diabetes and CVDs has been suggested by numerous researches highlighting the triggering effects of Western dietary pattern [4–7]. Obesity is associated with impaired acid-base balance; obesity lead to higher urinary calcium excretion and reduced urinary pH [8]. It has also been suggested that hydrogen ion accumulation due to acidogenic diets is associated with increased weight gain and obesity and that possibly meat and western diet is a cause of increased organic acid production and fatty acid oxidation in obese individuals; a condition which might be reversed in higher vegetables and fruits consumption [9]. High acidogenic contents of foods including meat, fish, cheese and lower alkaline content of diet including fruits and vegetables are the potential cause of endogenous acid production and elevated dietary acid load [10]. In fact, diet is responsible for more than 10-fold difference in endogenous acid production in different individuals [4]. The diet-induced acid load is estimated according to potential renal acid load (PRAL) and net-endogenous acid production (NEAP) according to information about ingested protein, potassium, calcium, phosphorous and magnesium [11]. The PRAL calculation is based on the formula first suggested by Remer et al [12] as follows: PRAL (mEq/d) = 0.4888 × protein intake (g/day) + 0.0366 × phosphorus (mg/day) -0.0205 × potassium (mg/ day) -0.0125 × calcium (mg/day) - 0.0263 × magnesium (mg/day). While NEAP is calculated based on the Frassetto et al suggested formula [13] as: Estimated NEAP (mEq/d) = [54.5 × protein intake (g/day) ÷ potassium intake (mEq/day)] - 10.2. These estimates are validated according to the estimated equivalents in the 24 hours urine measurement [12, 13]. Numerous studies are available reporting the association between metabolic risk factors with dietary acid load as either PRAL or NEAP or both of them [7, 11, 14–19]. The results of these studies are inconsistence; several reporting the positive association between metabolic risk factors [5, 6, 20] while others not [4, 21]. According to our literature review, only one meta-analysis was carried out evaluating the association between dietary acid load and risk of type two diabetes [22]. While no study is available summarizing the association between dietary acid load either as PRAL or NEAP with obesity indices, prevalence and serum lipids status. Therefore, in the current meta-analysis we summarize the results of studies evaluated the association between PRAL of NEAP with general or central obesity indices (e.g. BMI, WC, WHR), obesity prevalence, serum lipids including TG, TC, LDL, HDL in an updated systematic review and meta-analysis.

## Methods

### Search strategy

We performed a systematic search using PubMed, Scopus, Web of Sciences and Cochrane electronic databases to the studies evaluated the association between dietary acid load and general or central obesity, serum lipids and metabolic syndrome up to December 2018. No language restriction was applied. Moreover, hand-searching from reference lists of all relevant papers, previous reviews and meta-analyses was performed to cover all relevant publications. Strategy search was created using a combination of the MeSH (Medical Subject Headings) terms from the PubMed database and free text words were used. For each electronic database, search strategy was adopted. The PICO (patients, intervention, comparator and outcome) for studies’ selection is presented in Table 1. We used PICO model because it is one of the most widely used models for formulating clinical questions. The PICO model is one of the frequently used tools for structuring clinical research questions in connection with evidence syntheses. The Cochrane Handbook for Systematic Reviews of Interventions specifies using PICO as a model for developing a review question, thus ensuring that the relevant components of the question are well defined [23, 24].

**Table 1:** The PICO criteria used for the present systematic review.

PICO criteria	Description
Participants	General adult population
Exposure (Interventions)	Highest category of dietary acid load represented by higher scores of PRAL or NEAP
Comparisons	Lowest category of dietary acid load represented by higher scores of PRAL or NEAP
Outcome	BMI, WC, TG, LDL, TC, HDL, obesity prevalence
Study design	Observational studies with the design of cross-sectional, case control or cohort

### Selection and characteristics of the included studies

**Fig 1:**
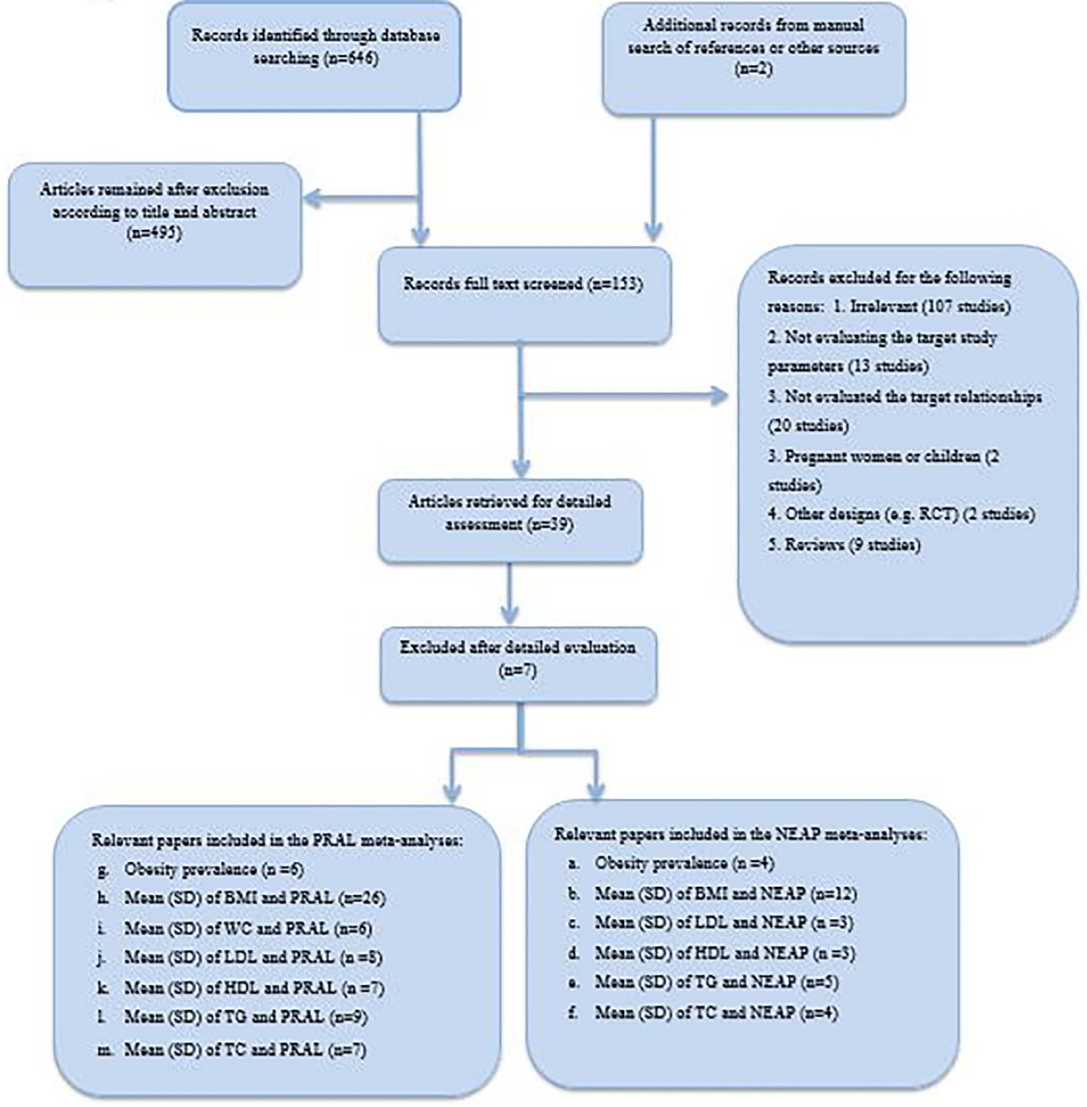
Flow diagram of study screening and selection process.

Our search obtained 646 potentially relevant articles from PubMed, Scopus, Cochrane and Google Scholar electronic databases. Thereafter 153 manuscripts were remained for full text screening after duplicate remove and exclusion after title and abstract reading. Totally, 121 manuscripts were excluded because of their irrelevant subject, inappropriate design, being reviews including meta-analysis or systematic reviews, conferences and seminars, not relevant age groups, not evaluating the association of studied parameters (dietary acid load, obesity, lipids and metabolic syndrome) or not measuring the routine dietary acid load. Accordingly 32 manuscripts were included in the systematic review. The Flow diagram of study screening and selection process is presented in Fig 1.

### Inclusion criteria

In the current systematic review and meta-analysis, observational studies with the design of cross-sectional, case control or cohort evaluating the association between dietary acid load and BMI, WC, WHR, obesity, central obesity, lipid profile and metabolic syndrome were included. According to our set of parameters, we conducted numerous meta-analyses. Dietary acid load-obesity or metabolic syndrome meta-analysis included the studies evaluated the odds ratio (OR), relative risk (RR) or prevalence of obesity or metabolic syndrome in the lowest versus highest dietary acid load categories. Accordingly, in dietary acid load–body mass index, dietary acid load–waist circumference or dietary acid load—serum lipids meta-analyses, the study must have reported the mean ± SD of body mass index or waist circumference or waist to hip ratio or serum lipids including triglyceride, cholesterol, low-density lipoprotein cholesterol, high density lipoprotein cholesterol in subjects in the highest versus lowest dietary acid load category as the reference group. For the search purpose, we used MESH (Medical Subject Heading) and non-MESH keywords including the following: (“dietary acid load” OR “dietary acid-based load”) AND (“body mass index” OR “BMI” OR “obesity” OR “central obesity” OR “serum lipids” OR “lipid profile” OR “triglyceride” OR “cholesterol” OR “LDL-cholesterol “low density lipoprotein cholesterol” OR “HDL” OR “high density lipoprotein cholesterol” OR “hypertension” OR “cardiovascular risk factors” OR “cardiometabolic risk factors” OR “metabolic syndrome” OR “diabetes”. The reviewed literatures were inserted into the EndNote software (version X8, for Windows, Thomson Reuters, Philadelphia, PA, USA). Consequently retrieved citations were merged, duplications were eliminated and the review process has been facilitated. Accordingly, titles and abstracts of all articles had been evaluated independently by three reviewers (MAF, LN, ZN). Articles not meeting the eligibility criteria were excluded. Moreover, the reference lists of relevant review article were also evaluated to include additional studies. Full-texts of relevant articles were retrieved if meeting the eligibility criteria, and wee re-evaluated. Any disagreements were discussed and resolved by consensus.

### Quality assessment

The methodological quality assessment of the included papers was performed by a nine-star Newcastle-Ottawa scale (NOS) for quality assessment of the cross-sectional, case-control and cohort studies. The 9-point NOS scale has scoring ranges from 0 to nine and is categorized into selection, comparability, and ascertaining of outcome. Studies with equal or more than 7 stars were categorized as high quality [25].

### Data collection and extraction

Data were collected according to a standard data extraction form gathering the information about the study characteristics including information about authors name, publication year, geographical area, study design; information about the population including participants age range, mean age of case and control group, number of case and controls, dietary assessment tool, setting, gender and the sample size and information about the adjusting for possible confounders the main findings and estimates of associations.

### Data synthesis and analysis

In the current meta-analysis, three meta-analysis approaches were used: the association between odds of obesity or metabolic syndrome and dietary acid load markers was analyzed by estimating the ORs and 95% confidence intervals by calculating the Ln of ORs and its standard error of mean (s.e.) as the effect size of the meta-analysis. Pooled OR [and 95% confidence interval (CI)] was estimated using a weighted random-effect model (the DerSimonian-Laird approach).

The comparison of the continuous variables including BMI, WC, WHR, TG, TC, LDL, HDL between highest versus lowest category of dietary acid load as reference group was performed by measuring the unstandardized mean differences as the effect size calculated by pooled estimate of weighted mean difference (WMD) with 95% confidence interval (CI), and the fixed effects and random effects models.

The prevalence of obesity in highest versus lowest dietary acid load categories was performed by re-calculating the proportions of interest from the relevant numerator and denominator. The overall proportions of interest were derived using meta-analysis techniques by metaprop command in the STATA and presented along with 95% confidence intervals (95% CI) calculated using a normal approximation. Cochran's Q test and I squared test was used to identify between-study heterogeneity; I^2^ < 25%, no heterogeneity; I^2^ = 25–50%, moderate heterogeneity; I^2^ > 50% large heterogeneity [26]. The heterogeneity was considered significant if either the Q statistic had p < 0.1 or I^2^ > 50%. Sensitivity analysis was used to explore the extent to which inferences might depend on a particular study or a number of publications. Subgroup analysis was performed to identify possible sources of heterogeneity, if required. Begg’s funnel plots was assessed to evaluate the publication bias followed by the Egger's regression asymmetry test and Begg's adjusted rank correlation for formal statistical assessment of funnel plot asymmetry. The data were analyzed using STATA version 13 (STATA Corp, College Station, TX, USA), and P-values less than 0.05 were considered as statistically significant.

## Results

### Description of the studies reported the dietary acid load as PRAL and NEAP with general and central obesity associations

From all of the systematically reviewed relevant papers (Table 2), totally 29 studies reported the association between PRAL and NEAP with obesity indices including BMI, WC, or the prevalence of general and central obesity [4–7, 11, 14–20, 27–43]. Twelve studies reported higher BMI or WC or the prevalence of general or central obesity in highest versus lowest category of dietary acid load indices [4, 14, 20, 27–29, 31, 32, 35, 37]. Examining the associations between diet-induced metabolic acidosis and cardio-metabolic risk factors among 27809 men and 36851 women of 45–75 years old, Akter S [20] reported higher BMI values in highest versus lowest PRAL quartiles among women (P <0.001) while among men an inverse relation between BMI trend among PRAL quartiles was reported. Other study by Kiefte-de Jong JC examining the association between energy-adjusted NEAP, PRAL and incident type 2 diabetes among 67433 women from the Nurses’ Health Study (NHS), 84310 women from the Nurses’ Health Study II (NHS- II) and 35743 men from the Health Professionals’ Follow-up Study (HPFS), reported higher BMI values in the top quintile versus low quintile of NEAP in baseline analysis of all three cohorts [32]. In other study by Kucharska AM et al [35], BMI, WC and the prevalence of overweight or obesity in highest NEAP tertile were higher than the lowest (P and P _trend_ < 0.05). While in PRAL categories, among men, no significant difference in the BMI and WC in the lowest versus highest PRAL tertiles was observed. The prevalence of overweight or obesity in the highest tertile of PRAL was lower than the lowest. Among women, BMI and WC and the prevalence of overweight or obesity in the highest tertile of PRAL were lower than the lowest. Murakami K [37] reported higher WC in highest quintile of PRAL among Japanese adults while no significant difference in BMI was reported. Totally, the unfavorable result of the higher BMI in lowest category of PRAL or NEAP was reported in three studies [11, 20, 35]. Other fifteen studies reported no association between dietary acid load and general or central obesity indices [5–7, 15, 17–19, 30, 33, 35, 36, 39–43].

**Table 2:** Characteristics of studies included in the systematic review owing to reporting the association between dietary acid load with general and central obesity indices and the prevalence of obesity.

First author	Year	Country	Study design	Sex	Age range	Sample size / Population	Number of cases / controls	Dietary assessment/index	Result	Adjusted variables	Quality of the study
Akter S [5]	2014	Japan	Cross-sectional	Both	18–70 y	2028/ working population	676/ 676	BDHQ/ PRAL, NEAP	No difference was found between BMI and PRAL or NEAP tertiles.	Age, sex	8
Akter S [6]	2016	Japan	Cross-sectional	Both	19–69 y	1732	433/433	BDHQ/ PRAL	No significant difference between BMI in different quartiles of PRAL.	-	6
Akter S [20]	2015	Japan	Cross-sectional	Men	45–75 y	27808	6952/6952	147-item FFQ /PRAL	BMI in lowest quartile of PRAL was higher than the highest (P = 0.01)	-	6
Akter S [20]	2015	Japan	Cross-sectional	Women	45–75 y	36851	9213/9213	147-item FFQ/PRAL	BMI in the highest quartile of the PRAL was higher than the lowest (P < 0.001)	-	6
Akter S [25]	2017	Japan	Cross-sectional	Both	45–75 y	92478	23119/23120	147-item FFQ/ PRAL	BMI in the highest quartiles of PRAL was significantly higher than the lowest (P <0.001)	-	6
Amodu A [4]	2013	USA	Cross-sectional	Both	≥ 20 y	13274	2490/2477	24-hour dietary recall questionnaire/NEAP	The prevalence of obesity in highest quartile of NEAP was significantly higher than the lowest (35.9 vs 24.8 P <0.001)	-	7
Bahadoran Z [26]	2015	Iran	Cross-sectional	Both	19–70 y	5620	1405/1405	147-item FFQ/ PRAL	BMI and WC and the prevalence of abdominal obesity in the highest quartile of PRAL was significantly higher than the lowest.	-	6
Banerjee T [27]	2018	USA	Cross-sectional	Both	21–84 y	3257	1074/1075	FFQ/ PRAL	BMI in the highest tertile of PRAL was significantly higher than the lowest (P = 0.0002)	-	7
Chan R [28]	2015	China	Cross-sectional	Both	≥ 65y	3122	780/779	FFQ/NEAP	No significant difference in the BMI in different quartiles of NEAP was reported.	-	7
Engberink MF [29]	2012	Netherland	Cross-sectional	Both	≥ 55 y	2241	747/747	FFQ/ PRAL	Mean BMI and the prevalence of overweight or obesity was higher in the highest versus lowest PRAL tertile.	-	6
Fagherazzi G [14]	2014	France	Cohort- baseline data for BMI	Women	40-65y	66485	16621/16622	208-item diet-history questionnaire/PRAL	Mean BMI and the prevalence of overweight or obesity was higher in the highest versus lowest PRAL quartile.	-	6
Faure AM [15]	2017	Switzerland	Cross-sectional	Men	≥ 60 y	117	29/29	110-item FFQ/PRAL	BMI was non-significantly higher in highest versus lowest PRAL quartiles.	-	6
Faure AM [15]	2017	Switzerland	Cross-sectional	Women	≥ 60 y	130	32/32	110-item FFQ/PRAL	BMI was non-significantly higher in highest versus lowest PRAL quartiles.	-	6
Gæde J [16]	2018	Denmark	Cohort (DCH)	Both	50–64 y	54651	10930/10931	192-item FFQ/PRAL	BMI was not significantly different between PRAL quintiles	-	5
Gæde J [16]	2018	Denmark	Cross-sectional (Inter99)	Both	30–60 y	5631	1126/1127	FFQ/PRAL	BMI was not significantly different between PRAL quintiles	-	6
Haghighatdoost F [17]	2015	Iran	Cross-sectional	Both	Mean age 66.8	547	274/273	FFQ/PRAL	No significant difference in the BMI, WC and the prevalence of obesity or abdominal obesity between PRAL groupings.	Protein, fat, cholesterol, fiber, whole refined grains, fruit, meat, potassium, phosphorus, beans, nuts, vegetables, BMI	5
Han E [11]	2016	Korea	Cross-sectional	Both	40–79 y	11601	4202/3859	One day 24-recall/ PRAL	BMI was slightly lower in highest versus lowest terrtile of PRAL. No difference in WC was observed.	-	7
Ikizler HO [18]	2016	USA	Cross-sectional	Both		63	21/21	3-day prospective food diaries/ NEAP	BMI was non-significantly higher in highest versus lowest NEAP tertile.	-	7
Iwase H [7]	2015	Japan	Cross-sectional	Both	Mean aged 65.7 ±9.3	149	74/75	Diet history questionnaire (DHQ)/ NEAP	No significant difference in BMI in highest versus lowest PRAL score groupings was observed.	-	6
Iwase H [7]	2015	Japan	Cross-sectional	Both	Mean aged 65.7 ±9.3	149	74/75	Diet history questionnaire (DHQ)/ NEAP	No significant difference in BMI in highest versus lowest PRAL score groupings was observed.	-	7
Jia T [19]	2015	Sweden	Cross-sectional	Both	≥ 70 y	861	215/ 215	7-day food records	No significant difference in BMI in highest versus lowest NEAP quartils was observed.		6
Kiefte-de Jong JC [30]	2017	USA	Cohort-NHS- median follow-up data	Women	30–55 y	121700	14974/ 11449	FFQ/ NEAP	Higher BMI in top quintile versus lowest quintile of NEAP observed.	Age	7
Kiefte-de Jong JC [30]	2017	USA	Cohort-NHS2- median follow-up data	Women	25–42 y	116430	13878/ 18030	FFQ/NEAP	Higher BMI in top quintile versus lowest quintile of NEAP observed.	Age	6
Kiefte-de Jong JC [30]	2017	USA	Cohort-HPFS- median follow-up data	Men	40–75 y	51529	7472/ 6428	FFQ/NEAP	Higher BMI in top quintile versus lowest quintile of NEAP observed.	Age	7
Ko BJ [31]	2017	Korea	Cross-sectional	Both	≥ 65 y	1369	342/343	FFQ/eNEAP	No significant difference in BMI between lowest and highest eNEAP quartiles was reported.	-	6
Krupp D [32]	2018	Germany	Cross-sectional	Both	18–79 y	7115	1358/1356	FFQ/PRAL	No significant difference in BMI between different PRAL quintiles was observed.	-	5
Kucharska AM [33]	2018	Poland	Cross-sectional	Men	≥ 20 y	2760	920/ 920	24h-recall/ NEAP	BMI and WC and the prevalence of overweight or obesity in the highest tertile of PRAL were higher than the lowest.	-	6
Kucharska AM [33]	2018	Poland	Cross-sectional	Women	≥ 20 y	3409	1136/ 1137	24h-recall/ NEAP	BMI and WC and the prevalence of overweight or obesity in the highest tertile of PRAL were higher than the lowest.	-	5
Kucharska AM [33]	2018	Poland	Cross-sectional	Men	≥ 20 y	2760	920/ 920	24h-recall/ PRAL	No significant difference in the BMI and WC in the lowest versus highest PRAL tertiles was observed. The prevalence of overweight or obesity in the highest tertile of PRAL was lower than the lowest.	-	7
Kucharska AM [33]	2018	Poland	Cross-sectional	Women	≥ 20 y	3409	1136/ 1137	24h-recall/ PRAL	BMI and WC and the prevalence of overweight or obesity in the highest tertile of PRAL were lower than the lowest.	-	5
Luis D [34]	2014	Sweden	Cross-sectional	Both	70–71 y	673	224/ 224	7-d food records/PRAL	No significant difference in BMI between tertiles of PRAL was observed.	-	6
Murakami K [35]	2008	Japan	Cross-sectional	Both	18–22 y	1136	227/ 227	DHQ/ PRAL	No significant difference in BMI between quintiles of PRAL was observed. WC in the highest quintile was significantly higher than the lowest.	Residential block, residential area size, survey year, PA current smoking,	8
Rebholz CM [36]	2015	USA	Cross-sectional	Both	45–64 y	15055	3011	FFQ/NEAP, PRAL	The prevalence of overweight or obesity in the highest quartile was significantly higher than the lowest (73.9 vs 59.5%)	-	6
Welch AA [37]	2007	UK	Cross-sectional	Men	42–82 y	6375	1275/ 1275	FFQ/ PRAL	No significant difference in BMI between quintiles of PRAL was observed.	-	6
Welch AA [37]	2007	UK	Cross-sectional	Women	42–82 y	8188	1639/1640	FFQ/ PRAL	No significant difference in BMI between quintiles of PRAL was observed.		6
Welch AA [38]	2013	UK	Cross-sectional	Women	18–79 y	2689	538/ 537	FFQ/ PRAL	No significant difference in BMI between quintiles of PRAL was observed.	-	5
Wynn E [39]	2008	Swiss	Cross-sectional	Women	≥ 75 y	401	133/134	FFQ/ NEAP	No significant difference in BMI between tertiles of NEAP was observed.	-	5
Hong Xu [40]	2016	Sweden	Cross-sectional	Women	45–84 y	36470	7294/ 7294	FFQ/ PRAL	No significant difference in BMI between quintiles of PRAL was observed.	-	5
Hong Xu [40]	2016	Sweden	Cross-sectional	Men	45–84 y	44957	9038/ 8984	FFQ/ PRAL	No significant difference in BMI between quintiles of PRAL was observed.	-	6
Hong Xu [41]	2016	Sweden	Cross-sectional	Both	70–71 y	911	304/ 303	7-d food records/PRAL	No significant difference in BMI between tertiles of PRAL was observed.	—	5

### Description of the studies reported the dietary acid load as PRAL and NEAP with serum lipids and CVD risk factors associations

From all of the systematically reviewed papers (Table 3) totally, seventeen studies reported the associations between PRAL and NEAP with cardiovascular disease (CVD) and serum lipids [4, 7, 11, 17, 19, 21, 28, 29, 31–37, 43, 44]. Higher concentrations of serum lipids including TG, LDL and lower HDL concentrations and higher prevalence of hypercholesterolemia in the highest versus lowest PRAL or NEAP categories was reported in six studies [7, 11, 17, 32, 35, 37]. In the study by Kiefte-de Jong JC et al among three cohort of NHS, NHS- II and HPFS, higher prevalence of hypercholesterolemia in the highest versus lowest quintiles of PRAL and NEAP was in both men and women was reported in all of three cohorts [32]. In other study by Kucharska AM [35] examining the association between dietary acid load and cardio-metabolic risk factors among polish adults, only serum TG tended to increase across tertiels of NEAP only among men; while no significant difference among women or across PRAL tertiles were observed [35]. On the other hand, three studies reported lower TC or higher HDL concentrations in highest versus lowest categories of PRAL or NEAP [21, 34, 35]. Other studies reported no significant difference between lipids across NEAP or PRAL categories [11, 28, 31, 33, 35]. The prevalence of CVD was reported among six studies [4, 19, 29, 31, 35, 36] while one study reporting an inverse association between the prevalence of CVD and NEAP quartile scores [4] and others reported no association. The prevalence of metabolic syndrome or the odds of it has also been reported in three studies [7, 11, 28]; Bahadoran et al [28] reported no difference in the prevalence of metabolic syndrome across quartiles of PRAL. In a cross-sectional population based study of 11601 general Korean population by Han et al higher prevalence of metabolic syndrome among highest versus lowest tertiles of PRAL was reported [11]. The third study, evaluated the association between PRAL, NEAP and metabolic risk factors among patients with T2DM, the prevalence of metabolic syndrome tend to decrease in PRAL or NEAP groupings while the odds of metabolic syndrome in the highest group of PRAL [OR = 2.22; CI: 1.04–4.83] or NEAP was higher than the lowest group as the reference group [OR = 2.61; CI: 1.25–5.55; P <0.001] after adjustment for age, sex, serum uric acid and creatinine, total energy intake, carbohydrate intake and sodium intake. In the study by Hong X et al no significant difference in the prevalence of hyperlipidemia between tertiles of PRAL was observed [7].

**Table 3:** Characteristics of studies included in the systematic review owing to reporting the association between dietary acid load with serum lipids and risk of CVD, hyperlipidemia and metabolic syndrome.

First author	Year	Country	Study design	Sex	Age range	Sample size / Population	Number of cases / controls	Dietary assessment/ index	Result	Adjusted variables	Quality of the study
Amodu A [4]	2013	USA	Cross-sectional	Both	≥ 20 years	13274	2490/2477	24-hour dietary recall questionnaire/ NEAP	The prevalence CVD in lowest quartile of NEAP was significantly higher than the highest (P < 0.001).	-	6
Bahadoran Z [26]	2015	Iran	Cross-sectional	Both	19–70 y	5620	1405/1405	147-item food-frequency questionnaire/ PRAL	No significant difference in TG and the prevalence of hypertriglyceridemia and low HDL and the prevalence of metabolic syndrome between quartiles was reported.	-	6
Banerjee T [27]	2018	USA	Cross-sectional	Both	21–84 y	3257	1074/1075	FFQ	No significant difference in the prevalence of CVD was reported.		6
Engberink MF [29]	2012	Netherland	Cross-sectional–baseline data	Both	≥ 55 y	2241/ general population	747/747	FFQ	No significant difference in the prevalence of CHD and the mean values of HDL and TC was observed.		7
Haghighatdoost F [17]	2015	Iran	Cross-sectional	Both	Mena age 66.8	547/ general population	274/273	FFQ/PRAL	TG was significantly higher in higher versus lowers PRAL score groupings.	Protein, fat, cholesterol, fiber, whole refined grains, fruit, meat, potassium, phosphorus, beans, nuts, vegetables, BMI	9
Han E [11]	2016	Korea	Cross-sectional	Both	40–79 y	11601/ general population	4202/3859	One day 24-recall/ PRAL	TG and the prevalence of metabolic syndrome in highest tertile was significantly higher than the lowest. TC, HDL and LDL were not different.		6
Iwase H [7]	2015	Japan	Cross-sectional	Both	Mean aged 65.7 ±9.3	149/ population with T2DM	74/75	Diet history questionnaire (DHQ)/ PRAL	LDL, TG and the odds of metabolic syndrome in highest group of PRAL was significantly higher than the lowest. The prevalence of metabolic syndrome in lowest group of PRAL score was higher than the highest.	age, sex, serum uric acid and creatinine, total energy intake, carbohydrate intake and sodium intake.	8
Iwase H [7]	2015	Japan	Cross-sectional	Both	Mean aged 65.7 ±9.3	149/ population with T2DM	74/75	Diet history questionnaire (HQ)/ NEAP	LDL and the odds of metabolic syndrome in highest group of PRAL was significantly higher than the lowest. No significant difference in TG was observed. The prevalence of metabolic syndrome in lowest group of PRAL score was higher than the highest	age, sex, serum uric acid and creatinine, total energy intake, carbohydrate intake and sodium intake.	8
Jia T [19]	2015	Sweden	Cross-sectional	Both	≥ 70 y	861/ general population	215/ 215	7-day food records/NEAP	No significant difference in the prevalence of CVD between NEAP quartiles was observed.	-	6
Kiefte-de Jong JC [30]	2017	USA	Cohort-NHS- median follow-up data	Both	30–55 y	121700/ general population	14974/ 11449	FFQ/ NEAP, PRAL	Higher prevalence of hypercholesterolemia in highest versus lowest quintile of NEAP was reported.	Age, energy intake, BMI, family history of diabetes, menopausal status, HTN and hypercholesterolemia, smoking, alcohol intake, PA, glycemic load, AHEI index, western dietary pattern	8
Kiefte-de Jong JC [30]	2017	USA	Cohort-NHS2- median follow-up data	Women	25–42 y	116430/ general population	13878/ 18030	FFQ/NEAP, PRAL	Higher prevalence of hypercholesterolemia in highest versus lowest quintile of NEAP was reported.	Age, energy intake, BMI, family history of diabetes, menopausal status, HTN and hypercholesterolemia, smoking, alcohol intake, PA, glycemic load, AHEI index, western dietary pattern	8
Kiefte-de Jong JC [30]	2017	USA	Cohort-HPFS- median follow-up data	Men	40–75 y	51529/ general population	7472/ 6428	FFQ/NEAP, PRAL	Higher prevalence of hypercholesterolemia in highest versus lowest quintile of NEAP was reported.	Age, energy intake, BMI, family history of diabetes, menopausal status, HTN and hypercholesterolemia, smoking, alcohol intake, PA, glycemic load, AHEI index, western dietary pattern	8
Ko BJ [31]	2017	Korea	Cross-sectional	Both	≥ 65 y	1369/ general population	342/343	FFQ/eNEAP	No significant difference in TG, TC, the prevalence of hyperlipidemia between lowest and highest eNEAP quartiles was reported.	-	7
Krupp D [32]	2018	Germany	Cross-sectional	Both	18–79 y	7115/ general population	1358/1356	FFQ/PRAL	TC was lower in fifth quintile compared with the first.	-	7
Kucharska AM [33]	2018	Poland	Cross-sectional	Men	≥ 20 y	2760/ general population	920/ 920	24h-recall/ NEAP	TG in highest tertile of NEAP was significantly higher than the lowest. No significant difference in other parameters.	Age, sex	8
Kucharska AM [33]	2018	Poland	Cross-sectional	Women	≥ 20 y	3409/ general population	1136/ 1137	24h-recall/ NEAP	No significant difference in TG, TC, LDL, HDL, the prevalence of hyperlipidemia and CVD between PRAL tertiles was observed.	Age, sex	7
Kucharska AM [33]	2018	Poland	Cross-sectional	Men	≥ 20 y	2760/ general population	920/ 920	24h-recall/ PRAL	No significant difference in TG, TC, LDL, the prevalence of hyperlipidemia and CVD between NEAP tertiles was observed. HDL in highest tertile was significantly higher than the lowest.	Age, sex	7
Kucharska AM [33]	2018	Poland	Cross-sectional	Women	≥ 20 y	3409/ general population	1136/ 1137	24h-recall/ PRAL	No significant difference in TG, TC, LDL and HDL, the prevalence of hyperlipidemia and CVD between PRAL tertiles was observed.	Age, sex	8
Luis D [34]	2014	Sweden	Cross-sectional	Both	70–71 y	673/ general population	224/ 224	7-d food records/PRAL	No significant difference in the prevalence of dyslipidemia, CVD between tertiles of PRAL was observed.	-	6
Moghadam SKH [42]	2016	Iran	Cross-sectional	Both	22–80 y	925/ general population	224	FFQ/ PRAL	No significant difference in LDL, HDL, TG at baseline between quartiles of PRAL was reported.	-	6
Moghadam SKH [42]	2016	Iran	Cohort	Both	22–80 y	925/ general population	224	FFQ/ PRAL, NEAP	No significant difference in LDL, HDL, TG after three years follow-up between quartiles of PRAL was reported.	Age, sex, BMI,PA, dietary energy, fat, carbohydrate, SFA, fiber	7
Murakami K [35]	2008	Japan	Cross-sectional	Both	18–22 y	1136/ general population	227/ 227	DHQ/ PRAL	Significantly higher values of TC, LDL between different quartiles of PRAL. No difference in HDL and TAG was observed.	Residential block, residential area size, survey year, PA current smoking, BMI, WC.	8
Berg EVD [21]	2012	Denmark	Cross-sectional	Both	≥ 18 y	707/ renal transplant patients	236/236	FFQ/NEAP	TC in the highest tertile of NEAP was significantly lower than the lowest. No difference in the TG, HDL between tertiles of NEAP was reported.	-	7
Hong Xu [41]	2016	Sweden	Cross-sectional	Both	70–71 y	911	304/ 303	7-d food records/PRAL	No significant difference in the prevalence of HLP between PRAL tertiles was observed.	Age, BMI, smoking status, PA, education, glucose disposal rate (M), CVD, HTN, HLP, energy-adjusted fiber, MUFA, PUFA, SFA, carbohydrate intake, eGFR, UAER	8

### Findings from meta-analysis of the prevalence of general obesity, central obesity and difference in BMI across lowest and highest dietary acid load categories

**Fig 2:**
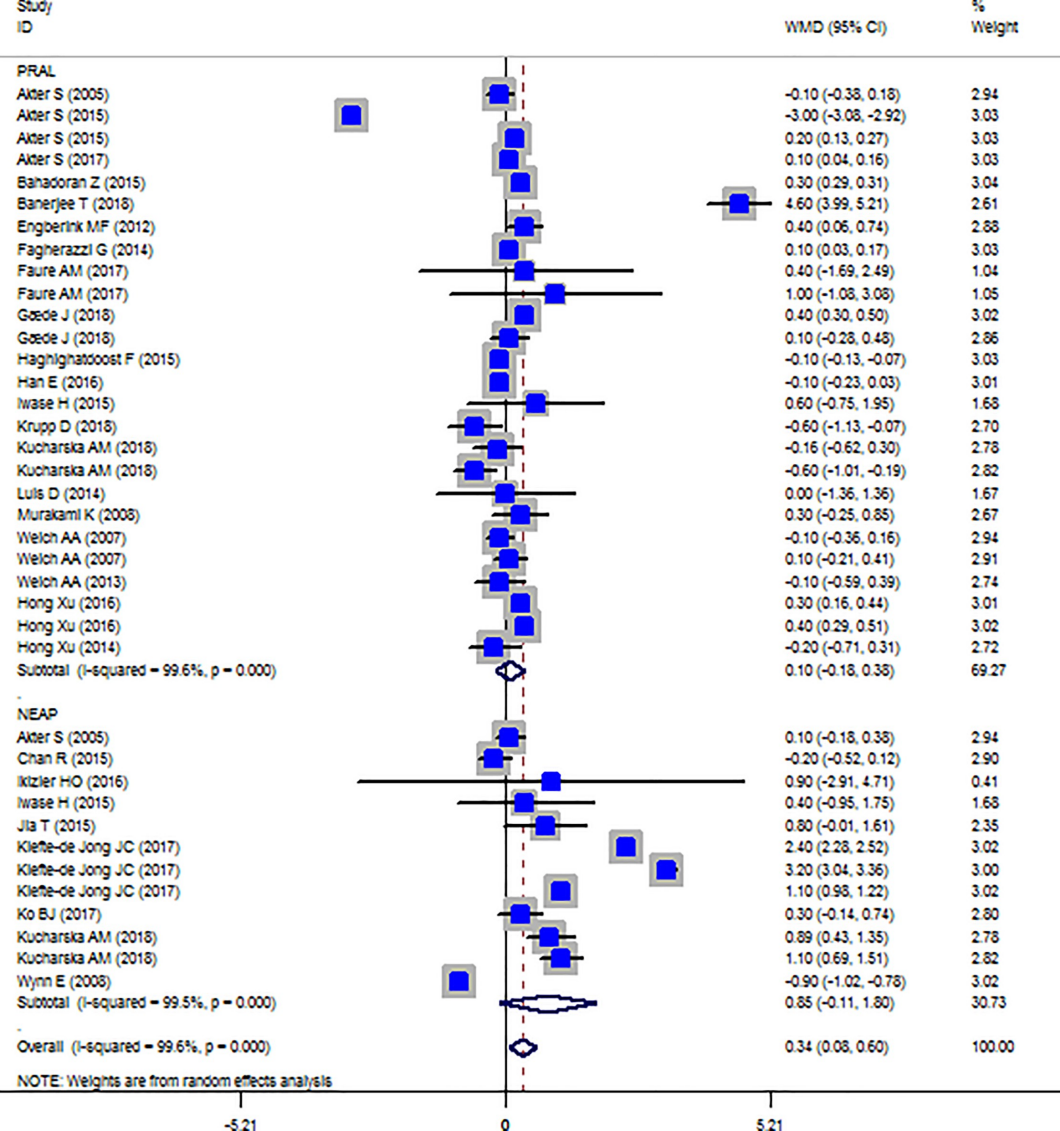
Forest plot illustrating obesity proportions in highest versus lowest PRAL categories.

The Forest plot of the studies included in the PRAL, NEAP and BMI meta-analysis are presented in Fig 2. No significant association between BMI and dietary acid load indices was observed (PRAL: WMD = 0.101, 95% CI = -0.179, 0.380; P = 0.481; NEAP: WMD = 0.845, 95% CI = -0.106, 1.797; P = 0.082). However, a great between-study heterogeneity was observed (PRAL: Heterogeneity chi-squared = 6716.43 (d.f. = 25), P < 0.001; I^2^ = 99.6%; Tau^2^ = 0.45; NEAP: Heterogeneity chi-squared = 2315.96 (d.f. = 11), P <0.001; I^2^ = 99.5%; Tau^2^ = 2.62). Sensitivity analysis showed that excluding the study by Akter et al [20] in men led to significance change in the effect size (WMD: 0.230; CI: 0.105, 0.354; P<0.001).

The results of subgroup analysis for the association between BMI with PRAL and NEAP are presented in Tables 4 and 5. Accordingly, for PRAL-BMI associations, country, dietary assessment tool and sample size are the possible source of heterogeneity whereas, in NEAP-BMI associations, country and dietary assessment tool are the source of heterogeneity. Subgroup analysis also revealed gender difference in the associations between PRAL, NEAP and BMI (Table 4). Higher PRAL scores were associated with increased BMI in females (WMD: 0.122; CI: -0.001, 0.245; P = 0.049) and not in men. While, NEAP and BMI association was significant only among men (WMD: 0.890; CI: 0.430, 1.350; P < 0.001).

**Table 4:** Results of subgroup analyses of the association between mean difference in BMI and PRAL according to study and participants’ characteristics.

Group	No. of studies	WMD (95% CI)	P _within group_	P _between group_	P _heterogeneity_	I^2^, %
**Total**	26	0.101–0.179, 0.380	0.481		<0.001	99.6%
**Country**						
USA	1	4.600 3.989 5.211	<0.001		-	-
Denmark	2	0.307 0.034 0.579	0.027	<0.001	0.132	56.0%
Japan	6	-0.345–1.645 0.956	0.604		<0.001	99.9%
France	1	0.100 0.030 0.170	0.005		-	-
UK	3	-0.027–0.213 0.158	0.772		<0.001	0.0%
Iran	2	0.100–0.292 0.492	0.616		<0.001	99.8
Korea	1	-0.100–0.233 0.033	0.141		-	-
Netherland	1	0.400 0.055 0.745	0.023		-	-
Switzerland	2	0.702–0.773 2.177	0.351		0.690	0.0%
Germany	1	-0.600–1.131–0.069	0.027		0.164	-
Poland	2	-0.392–0.822 0.039	0.074		0.112	48.5
Sweden	4	0.300 0.138 0.462	<0.001		0.597	50.0
**Continent**						
Europe/ USA	17	0.285 0.050 0.521	0.017	0.965	<0.001	94.1%
Asia	9	-0.251–0.759 0.257	0.333		<0.001	99.9%
**Dietary assessment tool**						
FFQ	17	0.185–0.179 0.549	0.320	<0.001	0.320	99.8%
DHQ	4	0.092 0.025 0.160	0.007		0.395	0.0%
Food Record	2	-0.175–0.652 0.301	0.471		0.471	0.0%
24-H-Recall	3	-0.248–0.549 0.052	0.105		0.079	60.6%
**Sample size**						
1000 <	6	-0.100–0.133–0.066	<0.001	<0.001	0.777	0.0%
1000–10000	12	0.309–0.066 0.683	0.107		<0.001	95.5%
>10000	8	-0.200–1.007 0.607	0.627		<0.001	99.9%
**Gender**						
Male	6	-0.444–2.261 1.373	0.632	<0.001	<0.001	99.8%
Female	8	0.122–0.001 0.245	0.049		0.002	69.4%
Both gender	12	0.297 0.111 0.483	0.002		<0.001	98.6%

Studies eligible for inclusion in the systematic review and meta-analysis

**Table 5:** Results of subgroup analyses of the association between mean difference in BMI and NEAP according to study and participants’ characteristics.

Group	No. of studies	WMD (95% CI)	P _within group_	P _between group_	P _heterogeneity_	I^2^, %
**Total**	12	0.845–0.106 1.797	0.082		<0.001	99.5%
**Country**						
USA	4	2.142 1.018 3.266	<0.001	<0.001	<0.001	99.4%
China	1	-0.200–0.523 0.123	0.224		-	-
Japan	2	0.112–0.159 0.384	0.418		0.670	0.0%
Swiss	1	-0.900–1.020–0.780	<0.001		-	-
Korea	1	0.300–0.135 0.735	0.177		-	0.0%
Poland	2	1.006 0.698 1.314	<0.001		0.506	-
Sweden	1	0.800–0.013 1.613	0.054		-	-
**Continent**						
USA	4	2.142 1.018 3.266	<0.001	<0.001	<0.001	99.4%
Europe	4	0.457–0.827 1.742	0.485		<0.001	97.9%
Asia	4	0.050–0.180 0.281	0.668		0.267	24.0%
**Dietary assessment tool**						
FFQ	6	0.986–0.393 2.365	0.161	<0.001	<0.001	99.8%
DHQ	2	0.112–0.159 0.384	0.418		0.670	0.0%
7 day-Food Record	1	0.800–0.013 1.613	0.054		-	-
24-H-Recall	2	1.006 0.698 1.314	<0.001		0.506	0.0%
3 day-Food Dairy	1	0.900–2.911 4.711	0.643		-	-
**Sample size**						
1000 <	4	0.100–1.132 1.331	0.874	<0.001	<0.001	85.4%
1000–10000	5	0.421–0.047 0.889	0.087		<0.001	87.5%
>10000	3	2.233 1.067 3.398	<0.001		<0.001	99.6%
**Gender**						
Male	1	0.890 0.430 1.350	<0.001	<0.001	-	-
Female	2	0.090–1.870 2.050	0.928		<0.001	98.8%
Both gender	9	1.036 0.185 1.886	0.017		<0.001	99.1%

Studies eligible for inclusion in the systematic review and meta-analysis

**Fig 3:**
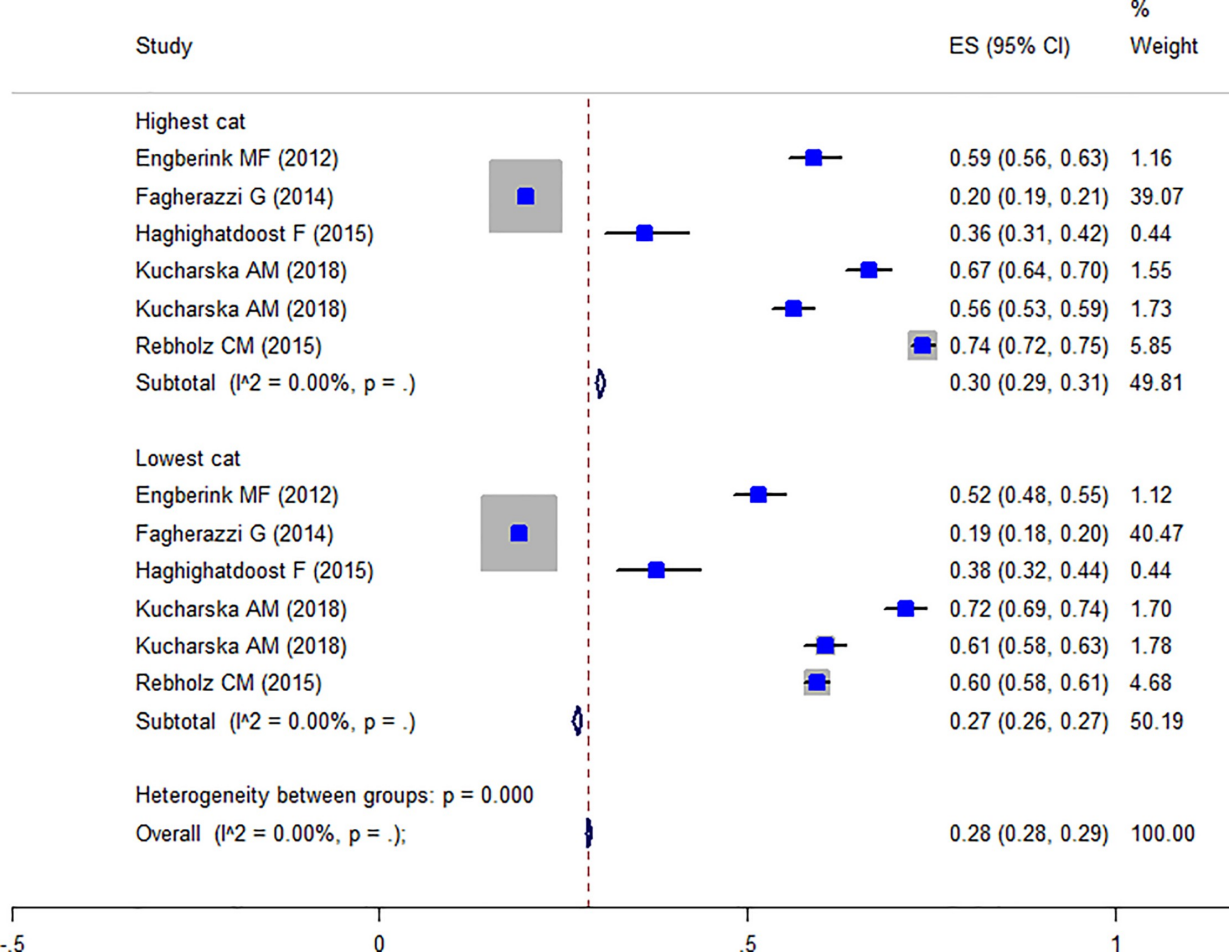
Forest plot illustrating obesity proportion in highest versus lowest NEAP categories.

**Fig 4:**
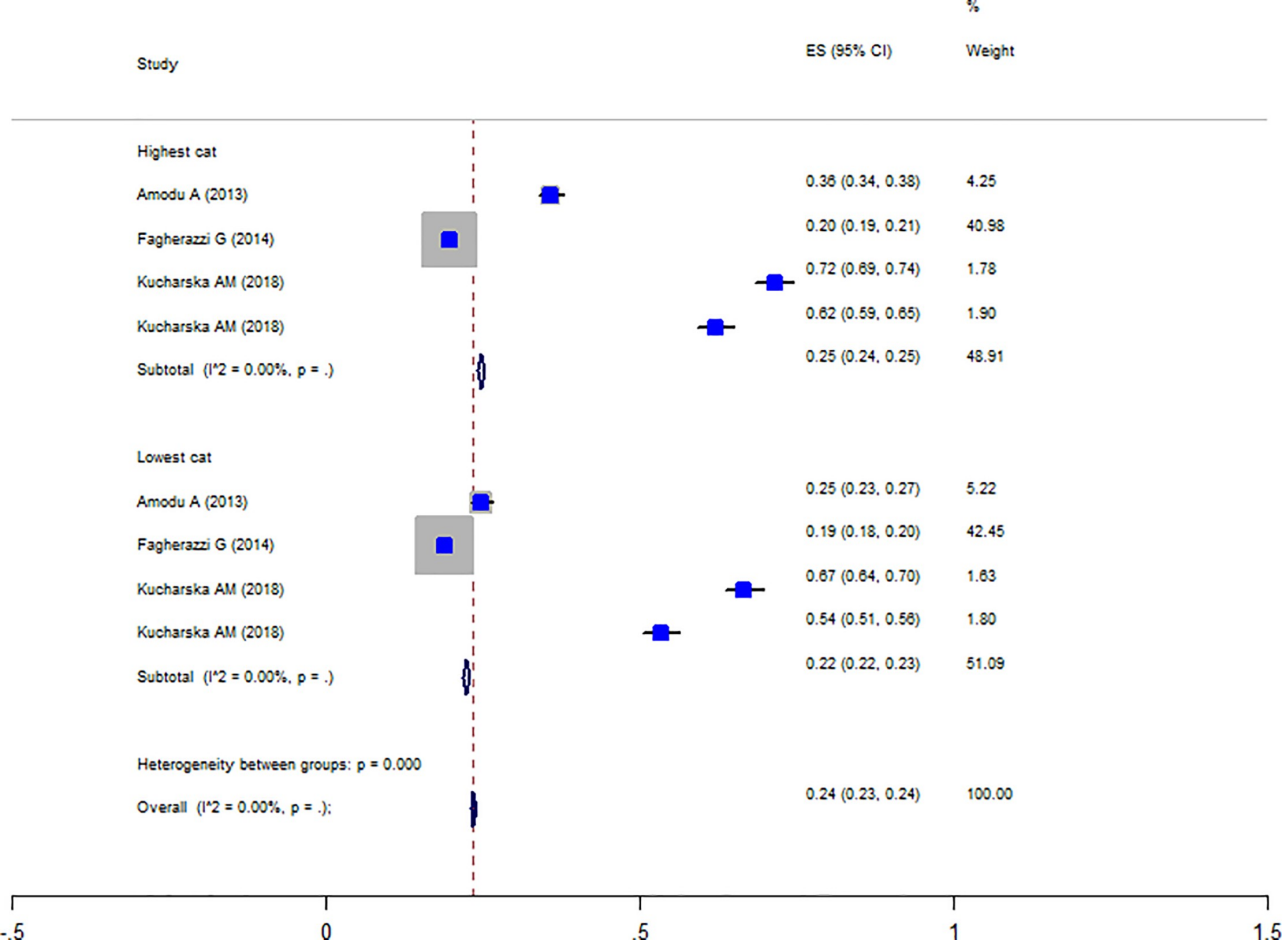
Forest plot illustrating weighted mean difference in BMI in highest versus lowest PRAL and NEAP.

**Fig 5:**
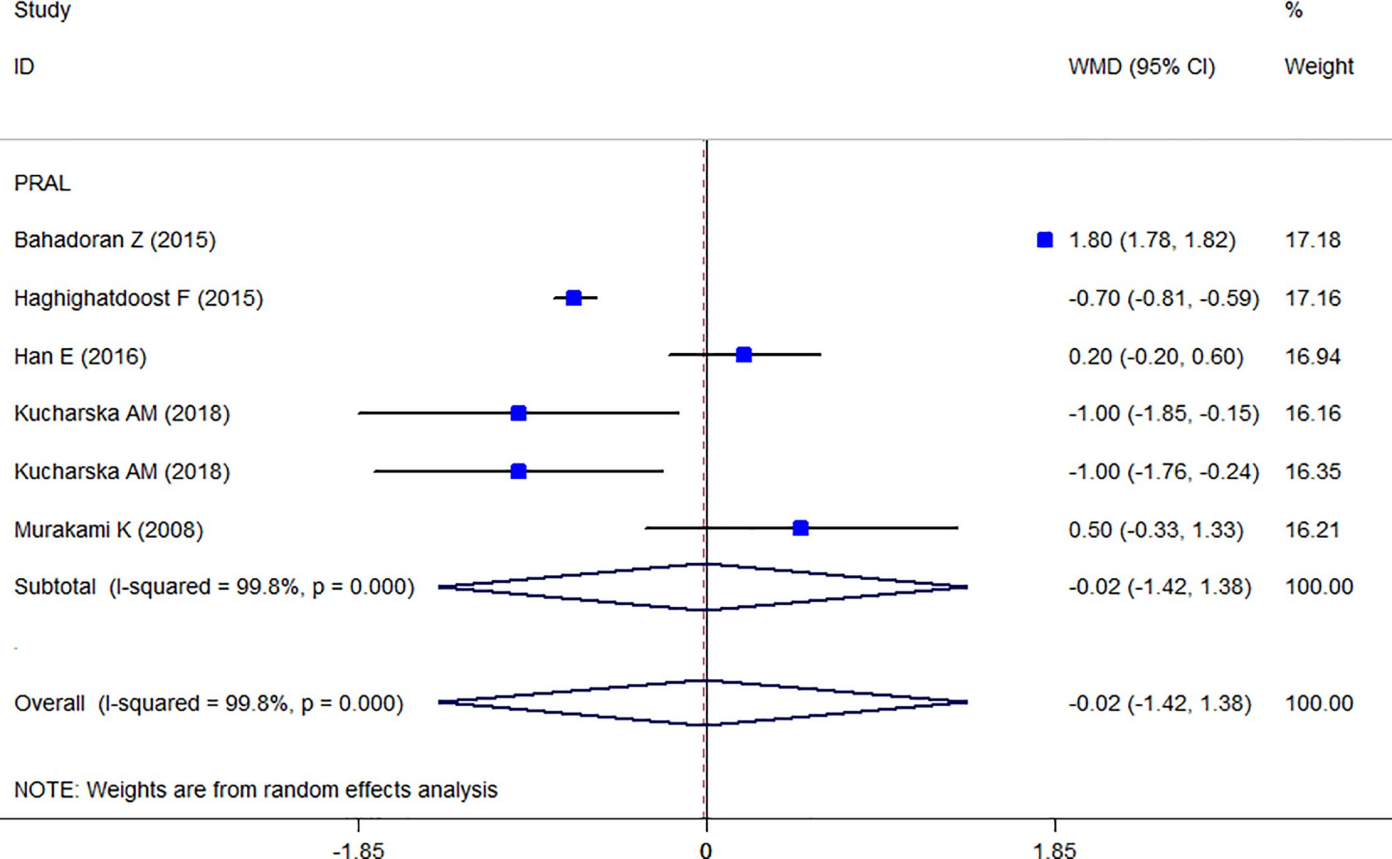
Forest plot illustrating weighted mean difference in WC in highest versus lowest PRAL.

Totally, five studies were reported the prevalence of obesity in different PRAL categories [14, 17, 31, 35, 38] and the Forest plot is presented in Fig 3. The prevalence of obesity in the highest PRAL category was 30% (CI: 0.29–0.31) and in the lowest category was 27% (CI: 0.26–0.27) with no evidence of heterogeneity. Moreover, three studies were also reported the prevalence of obesity in different NEAP categories [4, 14, 35] and the Forest plot (Fig 4) indicates that the prevalence of obesity in the highest NEAP category was 25% (CI: 0.24–0.24) and in the lowest category was 22% (CI: 0.22–0.23) with no evidence of heterogeneity. As shown, the higher prevalence of obesity in the highest scores of PRAL and NEAP had been reported. The prevalence of central obesity was identified in only two studies [17, 28] and therefore, no meta-analysis was performed. The association between waist circumferences as an indicator of central obesity with PRAL was reported in seven studies and the Forest plot (Fig 5) revealed no association (WMD: -0.021; CI:-1.422, 1.38, P = 0.977) with a great heterogeneity (Heterogeneity chi-squared = 2079.18 (d.f. = 5), P < 0.001; I^2^ = 99.8%; Tau^2^ = 2.97). In sensitivity analysis excluding the study by Wynn et al [41] revealed a significant association (WMD: 1.036; CI: 0.185, 1.886; P = 0.017). Table 6 presents the possible effects of country and continent on the heterogeneity. Among the reviewed studies only two studies reported the WC-NEAP associations and therefore were excluded from the meta-analysis [35, 37].

**Table 6:** Results of subgroup analyses of the association between mean difference in WC and PRAL according to study and participants’ characteristics.

Group	No. of studies	WMD (95% CI)	P _within group_	P _between group_	P _heterogeneity_	I^2^, %
**Total**	6	-0.021–1.422 1.38	0.977		<0.001	99.8%
**Country**						
Japan	1	0.500–0.330 1.330	0.238	<0.001	-	-
Iran	2	0.551–1.899 3.001	0.660		<0.001	99.9%
Korea	1	0.200–0.200 0.600	0.327		-	-
Poland	2	-1.000–1.568–0.432	0.001		1	0.0%
**Continent**						
Europe	2	-1.000–1.568–0.432	<0.001	<0.001	1	0.0%
Asia	4	0.450–1.248 2.149	0.603		<0.001	99.8%
**Dietary assessment tool**						
FFQ	2	0.551–1.899 3.001	0.660	<0.001	<0.001	99.9%
DHQ	1	0.500–0.330 1.330	0.238		0.003	82.5%
24-H-Recall	3	-0.544–1.457 0.368	0.242		-	-
**Sample size**						
<1000	1	-0.700–0.809–0.591	<0.001	<0.001	-	-
1000–5000	4	0.100–1.591 1.791	0.908		<0.001	97.1%
>5000	1	0.200–0.200 0.600	0.327		-	-
**Gender**						
Male	1	-1.000–1.850–0.150	0.021	<0.001	-	-
Female	2	-0.259–1.729 1.211	0.730		0.009	85.3%
Both gender	3	0.435–1.526 2.395	0.664		<0.001	99.9%

Studies eligible for inclusion in the systematic review and meta-analysis

### Findings from meta-analysis of mean TC, HDL-C, LDL-C, TG across different categories of dietary acid load

**Fig 6:**
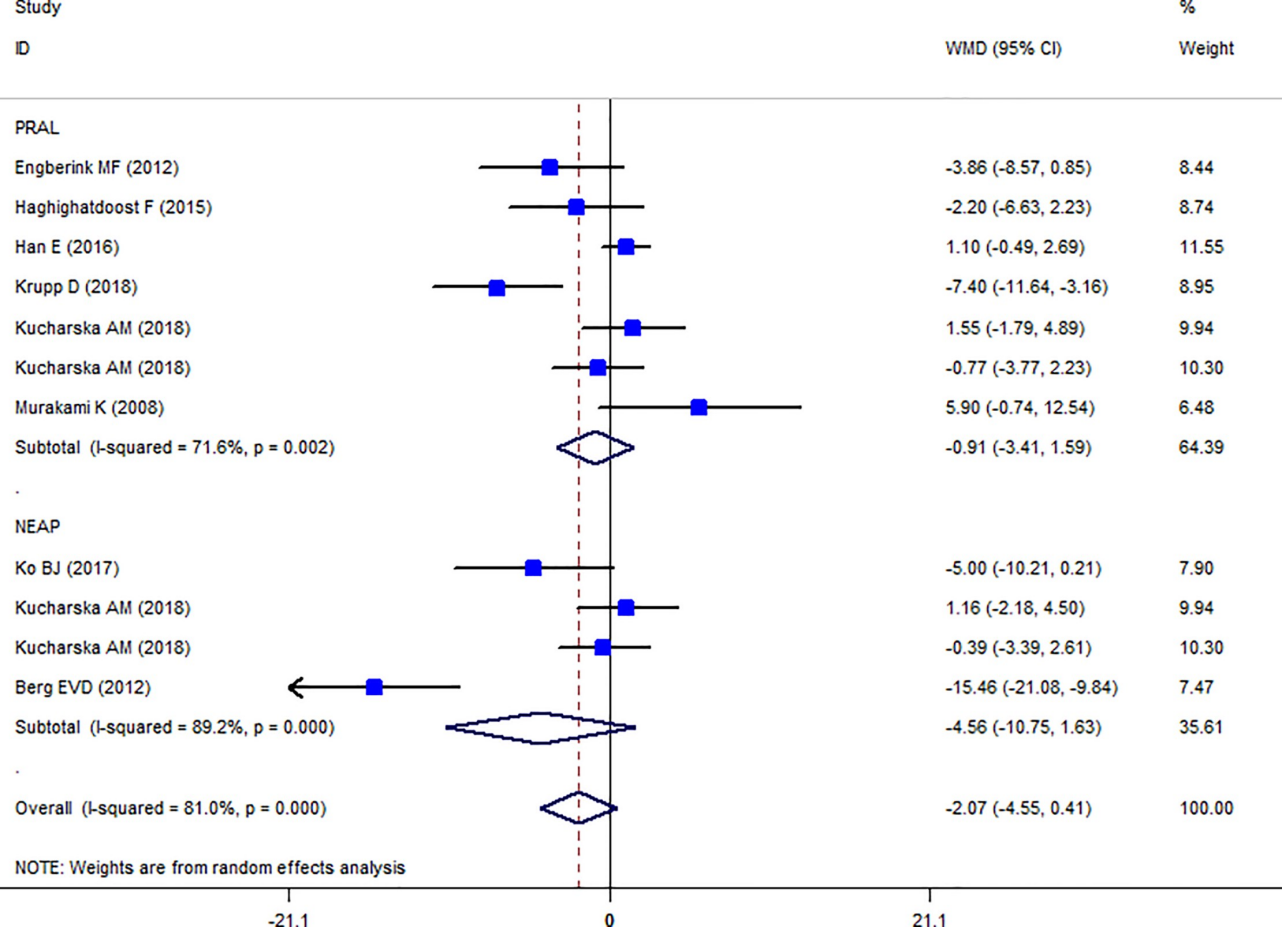
Forest plot illustrating weighted mean difference in TC in highest versus lowest PRAL and NEAP.

**Fig 7:**
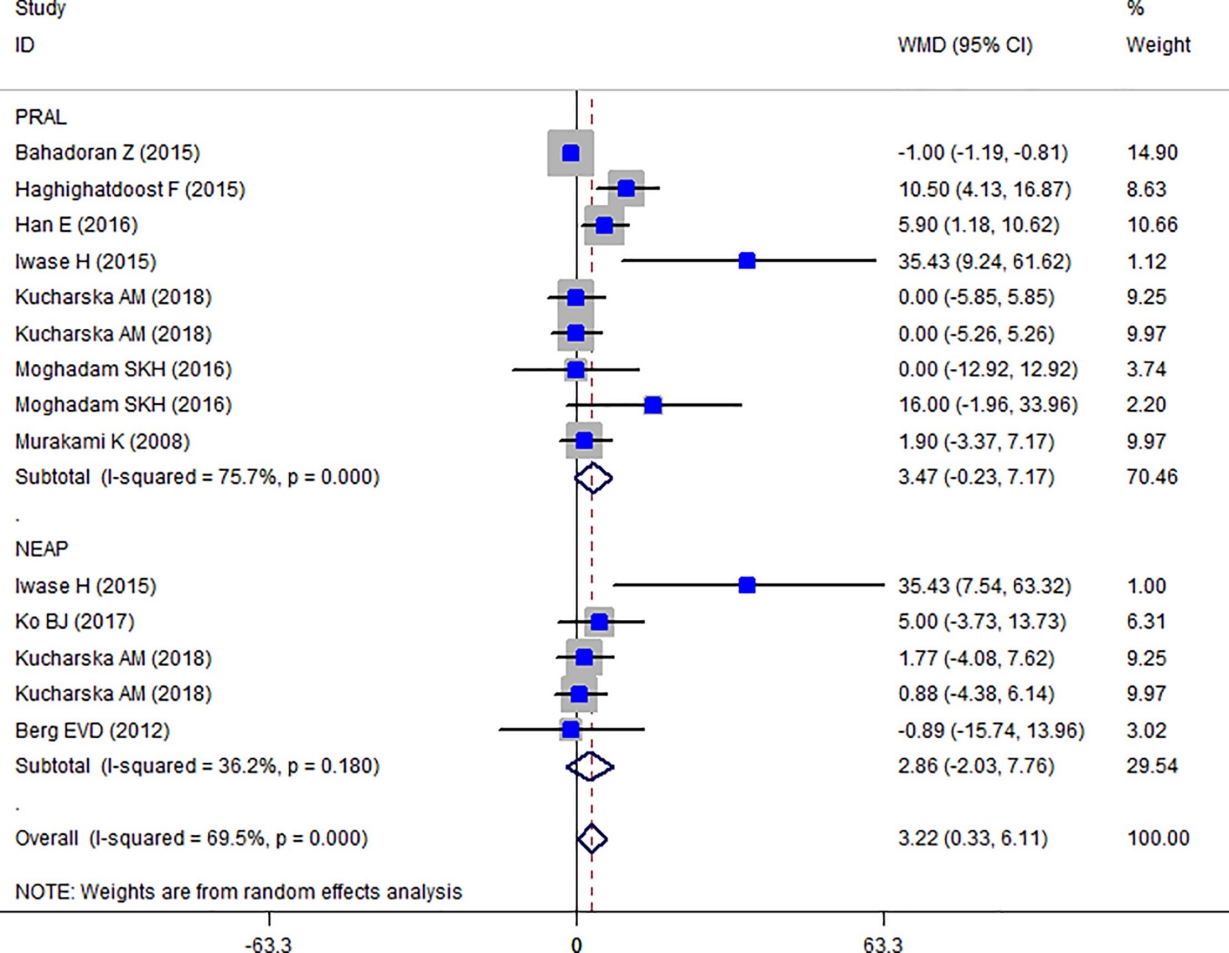
Forest plot illustrating weighted mean difference in TG in highest versus lowest PRAL and NEAP.

**Fig 8:**
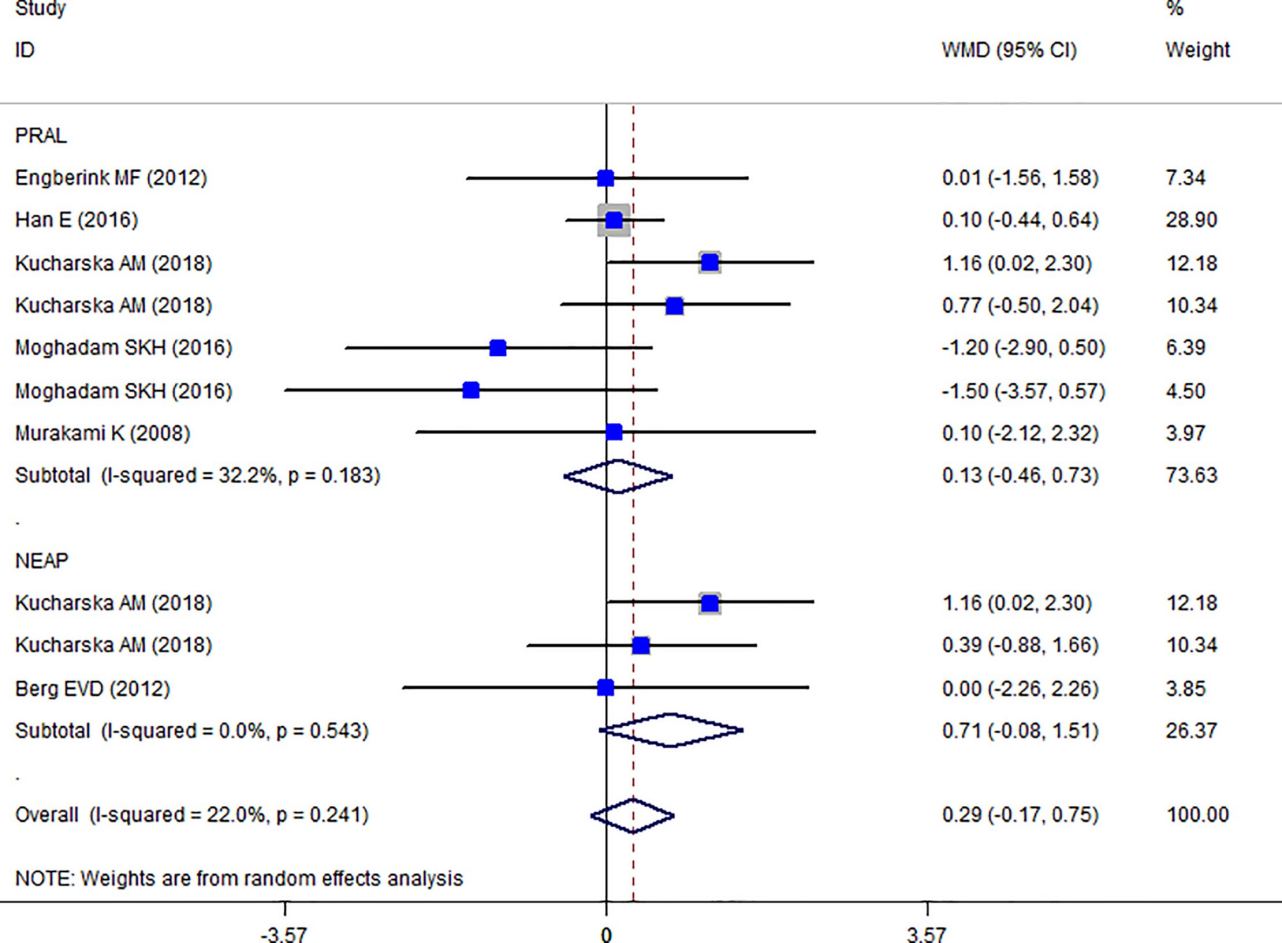
Forest plot illustrating weighted mean difference in HDL in highest versus lowest PRAL and NEAP.

**Fig 9:**
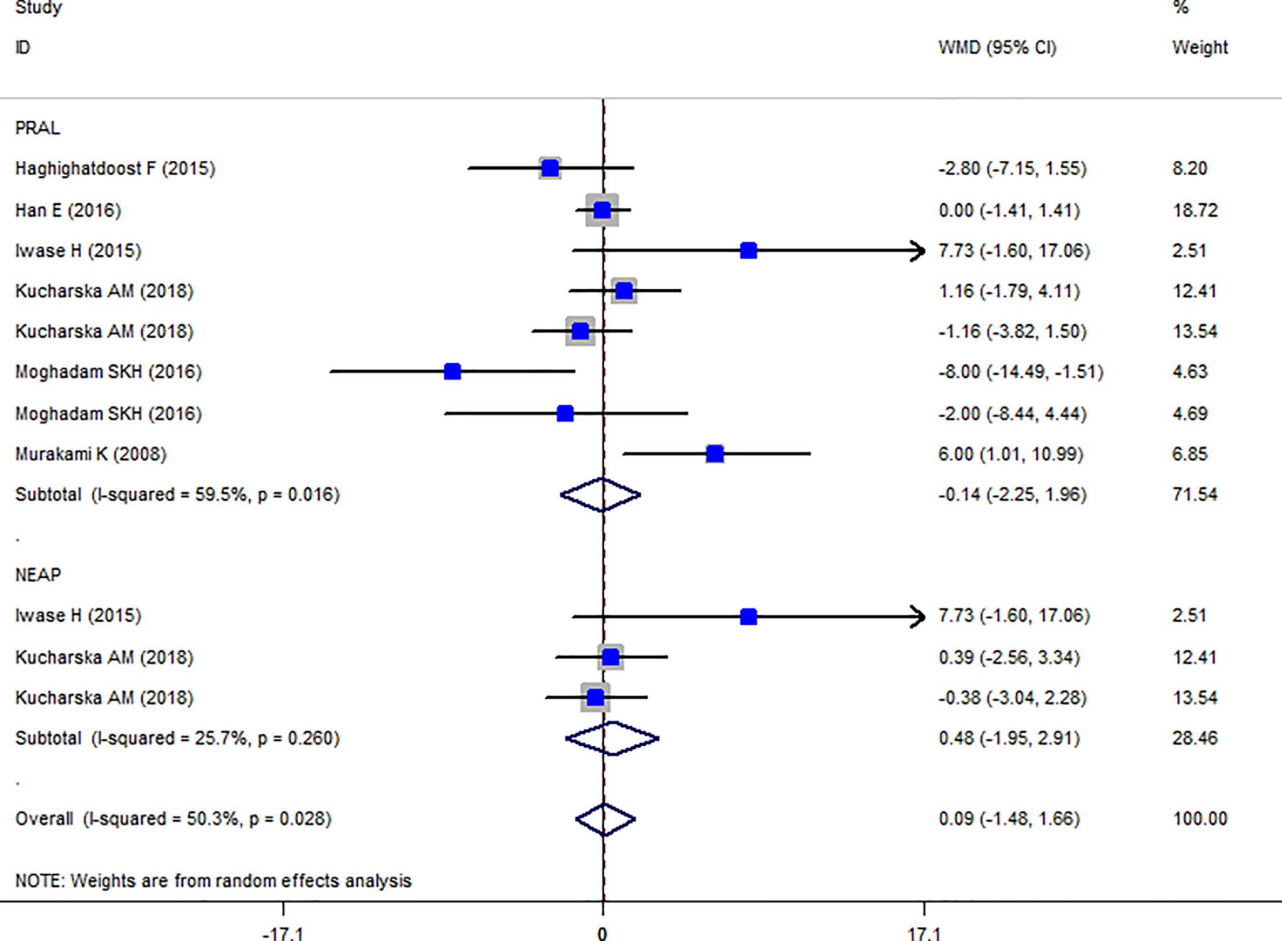
Forest plot illustrating weighted mean difference in LDL in highest versus lowest PRAL and NEAP.

The Forest plot of the effect of dietary acid load on serum TC (Fig 6) shows that no association between PRAL and NEAL with TC is present (PRAL: WMD = -0.911, 95% CI = -3.413, 1.590; P = 0.475; NEAP: WMD = -2.071, 95% CI = -4.549, 0.408; P = 0.149). A great heterogeneity was also observed (PRAL: Heterogeneity chi-squared = 21.16 (d.f. = 6), P = 0.002; I^2^ = 71.6%; Tau^2^ = 7.43; NEAP: Heterogeneity chi-squared = 27.81 (d.f. = 3), P <0.001; I^2^ = 89.2%; Tau^2^ = 34.92). Country dietary assessment tool and sample size might be the source of heterogeneity (Tables 7 and 8). Sensitivity analysis revealed no change in results. Association between PRAL, NEAP and serum TG is presented in Forest plot Fig 7 and accordingly, high PRAL scores are associated with 3.47 mg/dl increase in serum TG concentrations (WMD: 3.468; CI: -0.231, 7.166, P = 0.04) while no significant association between NEAP and TG was presented (WMD: 2.861; CI: -2.034, 7.756; P = 0.252). A great heterogeneity was also observed (PRAL: Heterogeneity chi-squared = 32.92 (d.f. = 8), P < 0.001; I^2^ = 75.7%; Tau^2^ = 17.94; NEAP: Heterogeneity chi-squared = 6.27 (d.f. = 4), P = 0.180; I^2^ = 36.2%; Tau^2^ = 10.50). The results of subgroup analysis are presented in Tables 9 and 10. The Forest plot of the association between PRAL, NEAP and serum HDL concentrations are presented in Fig 8. Accordingly, no effects of PRAL, NEAP and HDL was reported with minor heterogeneity (PRAL: WMD = 0.134; CI: -0.46, 0.728; P = 0.658; NEAP: WMD = 0.715, 95% CI = -0.081, 1.51; P = 0.078). The heterogeneity was minor (PRAL: Heterogeneity chi-squared = 8.84 (d.f. = 6), P = 0.183; I^2^ = 32.2%; Tau^2^ = 0.194; NEAP: Heterogeneity chi-squared = 1.22 (d.f. = 2), P = 0.543; I^2^ = 0.0%; Tau^2^ <0.001). In the case of the PRAL, NEAP and serum LDL concentrations also no association was observed (PRAL: WMD = -0.144; CI: -2.251, 1.96; P = 0.893; NEAP: WMD = 0.480, 95% CI = -1.954, 2.913; P = 0.669). The heterogeneity was meaningful for the PRAL-LDL meta-analysis (PRAL: Heterogeneity chi-squared = 17.29 (d.f. = 7), P = 0.016; I^2^ = 59.5%; Tau^2^ = 4.531; NEAP: Heterogeneity chi-squared = 2.69 (d.f. = 2), P = 0.260; I^2^ = 25.7%; Tau^2^ = 1.257; Fig 9, Table 11).

**Table 7:** Results of subgroup analyses of the association between mean difference in TC and PRAL according to study and participants’ characteristics.

Group	No. of studies	WMD (95% CI)	P _within group_	P _between group_	P _heterogeneity_	I^2^, %
**Total**	6	-0.911, 3.413 1.590	0.475		0.002	71.6
**Country**						
Netherland	1	-3.860–8.566 0.846	0.108	<0.001	-	-
Iran	1	-2.200–6.634 2.234	0.331		-	-
Korea	1	1.100–0.486 2.686	0.174		-	-
Germany	1	-7.400–11.642–3.158	0.001		-	-
Japan	1	5.900–0.741 12.541	0.082		-	-
Poland	2	0.271–1.990 2.533	0.814		0.311	2.6%
**Continent**						
Europe	4	-2.390–6.096 1.315	0.206	0.021	0.008	74.5%
Asia	3	0.974–2.230 4.178	0.551		0.128	51.3%
**Dietary assessment tool**						
FFQ	3	-4.564–7.658–1.470	0.004	<0.001	0.235	30.9%
DHQ	1	5.900–0.741 12.541	0.082		-	-
24-H-Recall	3	0.821–0.472 2.113	0.213		0.501	0.0%
**Sample size**						
<2000	2	1.457–6.443 9.358	0.718	0.066	0.047	74.7%
2000–10000	4	-2.390–6.096 1.315	0.206		0.008	74.5%
>10000	1	1.100–0.486 2.686	0.174		-	-
**Gender**						
Male	1	1.550–1.786 4.886	0.362	0.511	-	-
Female	2	0.361–2.374 3.096	0.796		0.073	68.9%
Both gender	4	-0.474–1.823 0.876	0.492		0.001	81.9%

Studies eligible for inclusion in the systematic review and meta-analysis

**Table 8:** Results of subgroup analyses of the association between mean difference in TC and NEAP according to study and participants’ characteristics.

**Group**	**No. of studies**	**WMD (95% CI)**	**P**_**within group**_	**P**_**between group**_	**P**_**heterogeneity**_	I^2^, %
**Total**	4	-2.071 4.549, 0.408	0.149		<0.001	89.2%
**Country**						
Korea	1	-5.000–10.210 0.210	0.060	<0.001	-	-
Denmark	1	-15.460–21.076–9.844	<0.001		0.498	0.0%
Poland	2	0.303–1.928 2.534	0.790		-	-
**Continent**						
Europe	3	-4.520–12.514 3.473	0.268	0.216	<0.001	92.5%
Asia	1	-5.000–10.210 0.210	0.060		-	-
**Dietary assessment tool**						
FFQ	2	-0.316–0.662 0.030	0.073	<0.001	0.003	88.3%
24-H-Recall	2	0.008–0.053 0.069	0.790		0.498	0.0%
**Sample size**						
<1500	2	-0.316–0.662 0.030	0.073	<0.001	0.003	88.3%
>1500	2	0.008–0.053 0.069	0.790		0.498	0.0%
**Gender**						
Male	1	0.032–0.060 0.123	0.496	<0.001	-	-
Female	1	-0.011–0.093 0.072	0.799		-	-
Both gender	2	-0.316–0.662 0.030	0.073		0.003	88.3%

Studies eligible for inclusion in the systematic review and meta-analysis

**Table 9:** Results of subgroup analyses of the association between mean difference in TG and PRAL according to study and participants’ characteristics.

Group	No. of studies	WMD (95% CI)	P _within group_	P _between group_	P _heterogeneity_	I^2^, %
**Total**	9	3.468–0.231, 7.166	0.04		0.001	75.7%
**Country**						
Iran	4	4.990–3.397 13.377	0.244	0.012	0.001	81.2%
Korea	1	5.900 1.180 10.620	0.014		-	-
Japan	2	16.111–16.365 48.586	0.331		0.014	83.5%
Poland	2	0.000–3.912 3.912	1.000		1.000	0.0%
**Dietary assessment tool**						
FFQ	4	4.990–3.397 13.377	0.244	0.025	<0.001	81.2%
DHQ	2	16.111–16.365 48.586	0.331		0.014	83.5%
24-H-Recall	3	2.205–1.841 6.251	0.286		0.169	43.8%
**Sample size**						
<1000	5	8.291 0.495 16.086	0.037	<0.001	0.025	64.0%
1000–5000	2	0.000–3.912 3.912	1.000		1.000	0.0%
>5000	2	2.030–4.681 8.742	0.553		0.004	87.8%
**Gender**						
Male	1	0.000–5.849 5.849	1.000	0.568	-	-
Female	2	0.949–2.773 4.671	0.617		0.617	0.0%
Both gender	6	6.719 0.035 13.403	0.049		<0.001	84.1%

Studies eligible for inclusion in the systematic review and meta-analysis

**Table 10:** Results of subgroup analyses of the association between mean difference in TG and NEAP according to study and participants’ characteristics.

Group	No. of studies	WMD (95% CI)	P _within group_	P _between group_	P _heterogeneity_	I^2^, %
**Total**	5	2.861–2.034, 7.756	0.252		0.180	36.2%
**Country**						
Denmark	1	-0.890–15.739 13.959	0.906	0.107	-	-
Korea	1	5.000–3.734 13.734	0.262		-	-
Japan	1	35.430 7.536 63.324	0.013		-	-
Poland	2	1.278–3.752 6.309	0.618		0.863	0.0%
**Dietary assessment tool**						
FFQ	2	3.486–4.043 11.015	0.364	0.06	0.503	0.0%
DHQ	1	35.430 7.536 63.324	0.013		-	-
24-H-Recall	2	1.278–3.752 6.309	0.618		0.863	0.0%
**Sample size**						
<1500	3	5.655–1.614 12.924	0.127	0.332	0.076	61.1%
>1500	2	1.278–3.752 6.309	0.618		0.863	0.0%
**Gender**						
Male	1	1.770–5.751 9.291	0.645	0.615	-	-
Female	1	0.880–5.887 7.647	0.799		-	-
Both gender	3	8.403–5.925 22.730	0.250		0.076	61.1%

Studies eligible for inclusion in the systematic review and meta-analysis

**Table 11:** Results of subgroup analyses of the association between mean difference in LDL and PRAL according to study and participants’ characteristics.

Group	No. of studies	WMD (95% CI)	P _within group_	P _between group_	P _heterogeneity_	I^2^, %
**Total**	8	0.144–2.251, 1.96	0.893		0.016	59.5
**Country**						
Iran	3	-3.862–7.122–0.601	0.020	0.003	0.348	5.3%
Korea	1	0.000–1.414 1.414	1.000		-	-
Japan	2	6.385 1.986 10.783	0.004		0.749	0.0%
Poland	2	-0.093–2.359 2.173	0.936		0.252	23.8%
**Dietary assessment tool**						
FFQ	3	-3.862–7.122–0.601	0.020	<0.001	0.348	5.3%
DHQ	2	6.385 1.986 10.783	0.004		0.749	0.0%
24-H-Recall	3	-0.041–1.191 1.108	0.944		0.517	0.0%
**Sample size**						
<1000	4	-2.080–7.087 2.926	0.415	0.168	0.060	59.5%
1000–10000	3	1.453–2.017 4.924	0.412		0.043	68.3%
>10000	1	0.000–1.414 1.414	1.000		-	-
**Gender**						
Male	1	1.160–1.792 4.112	0.441	0.538	-	-
Female	2	2.096–4.892 9.084	0.441		0.013	83.8%
Both gender	5	-1.476–4.839 1.887	0.390		0.042	59.5%

Studies eligible for inclusion in the systematic review and meta-analysis.

### Publication bias

**Fig 10:**
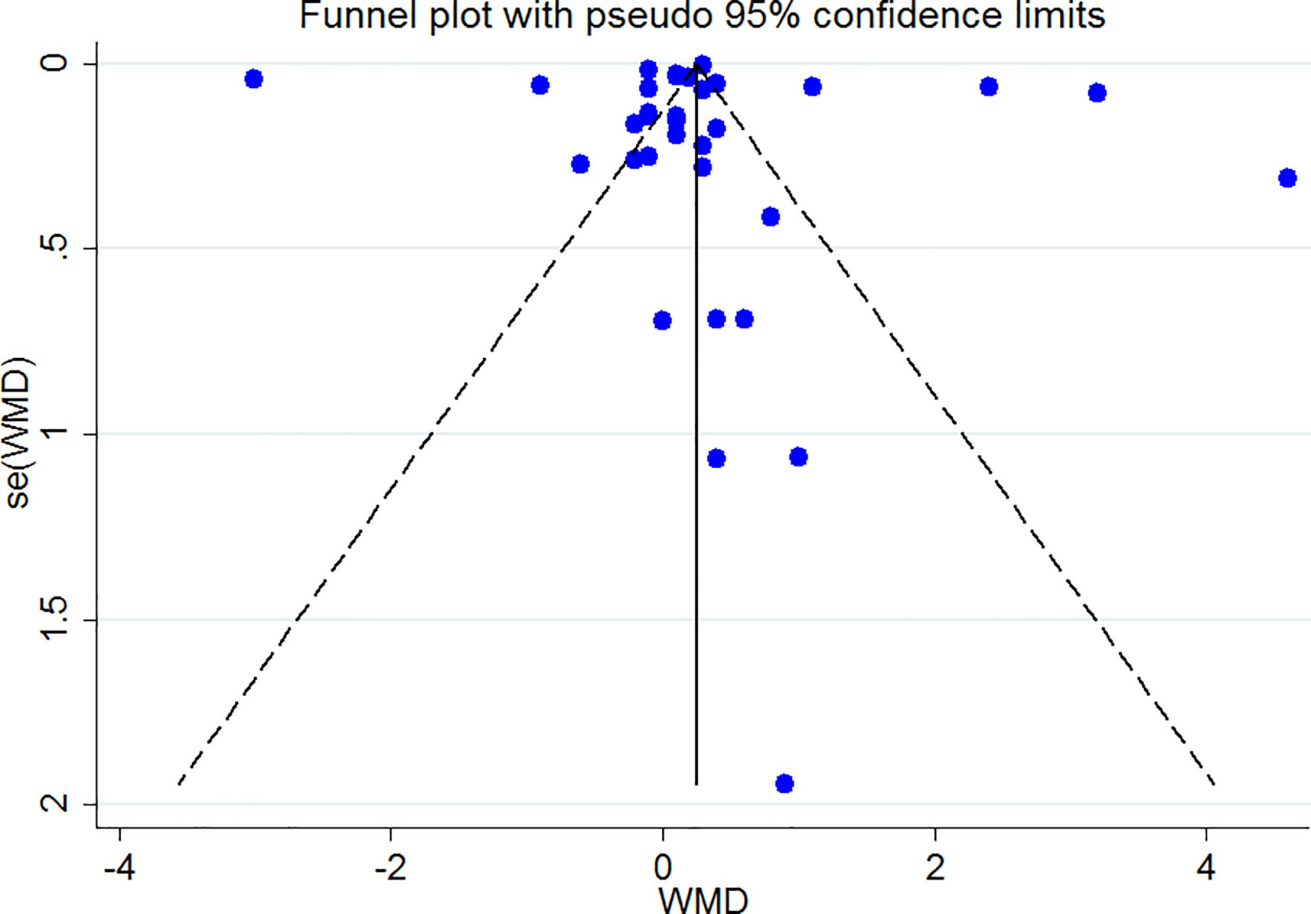
Begg's funnel plots (with pseudo 95% CIs) of the WMD versus the se (WMD) of the association between BMI, PRAL and NEAP.

**Fig 11:**
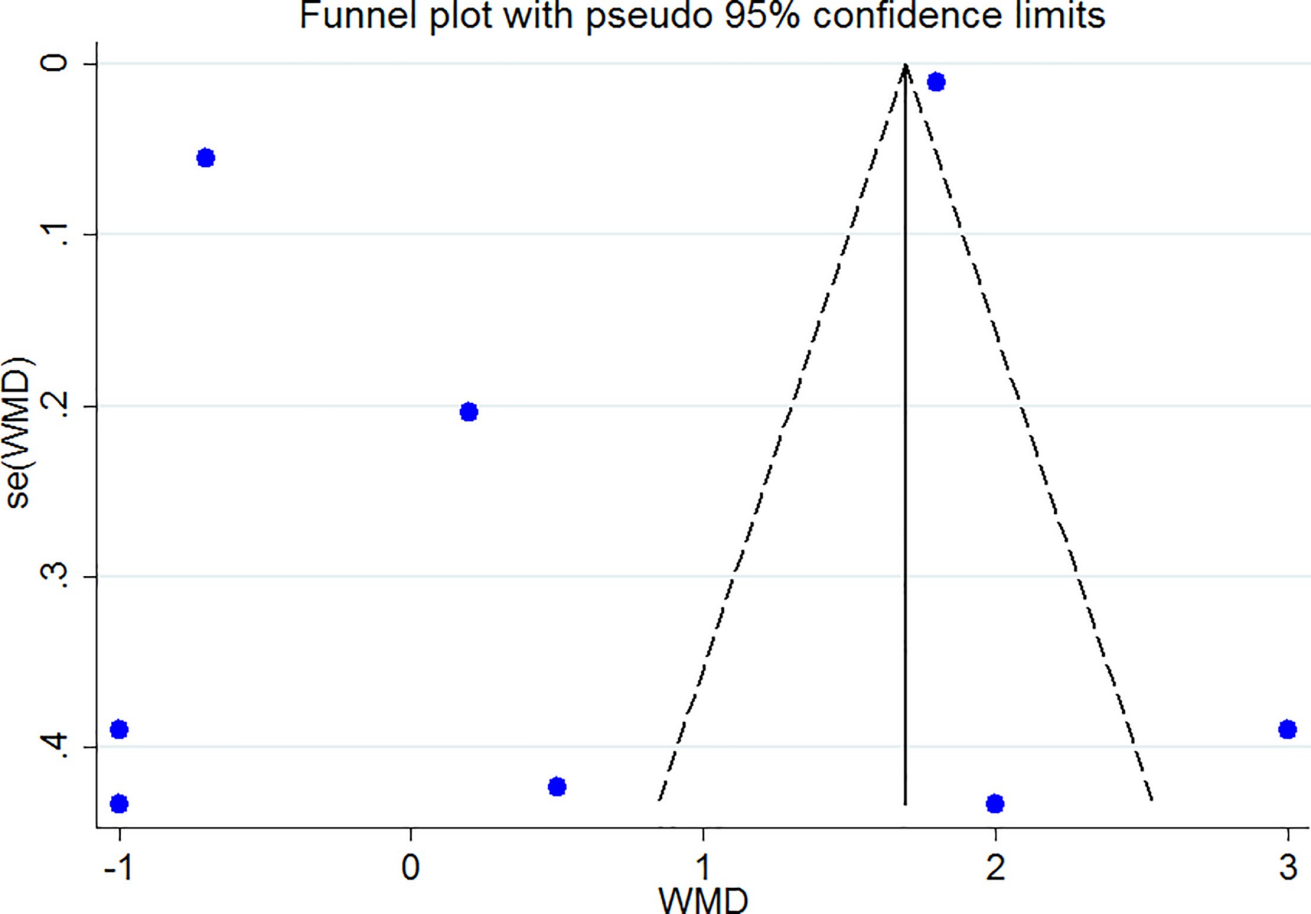
Begg's funnel plots (with pseudo 95% CIs) of the WMD versus the se (WMD) of the association between WC, PRAL and NEAP.

**Fig 12:**
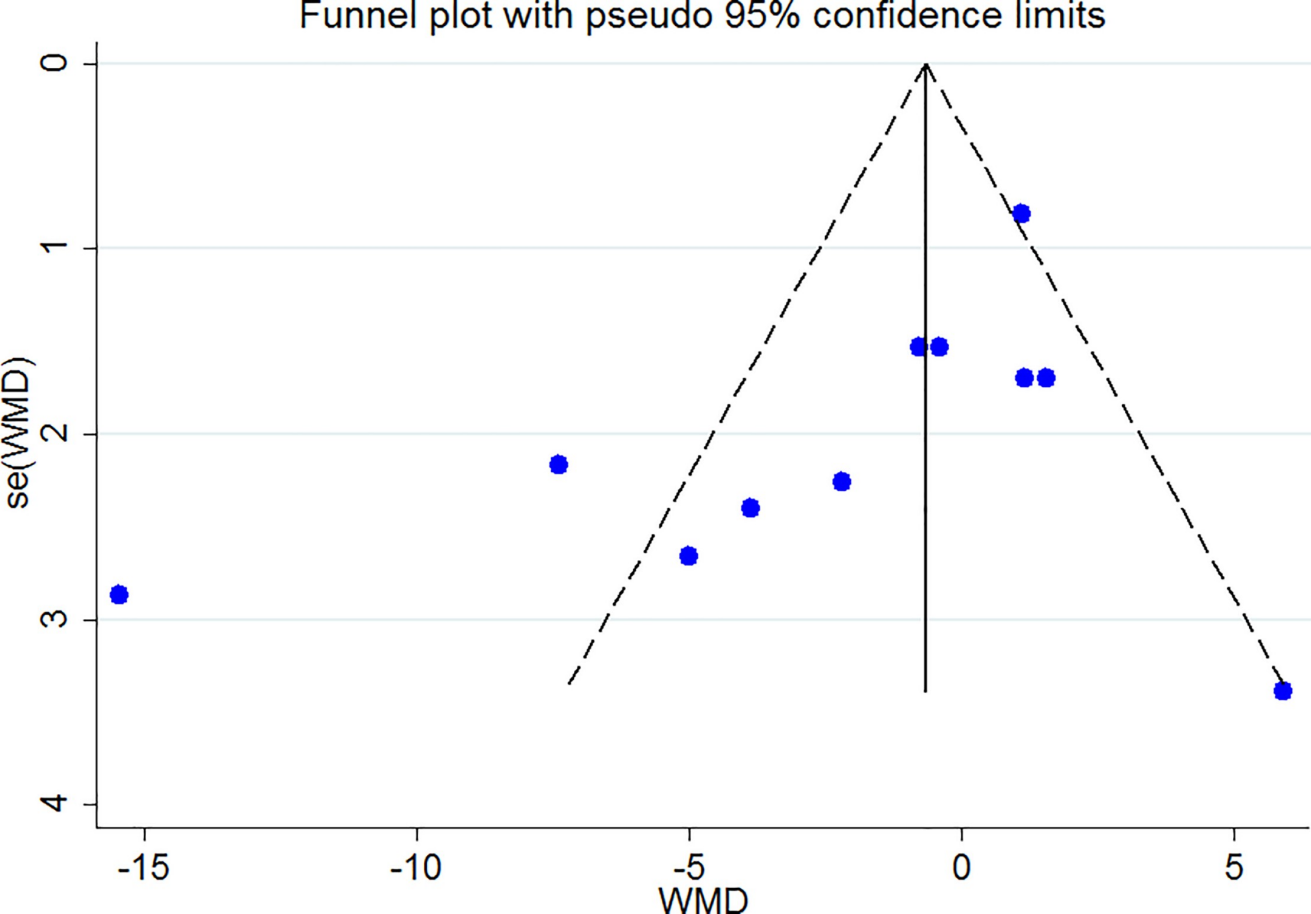
Begg's funnel plots (with pseudo 95% CIs) of the WMD versus the se (WMD) of the the association between TC, PRAL and NEAP.

**Fig 13:**
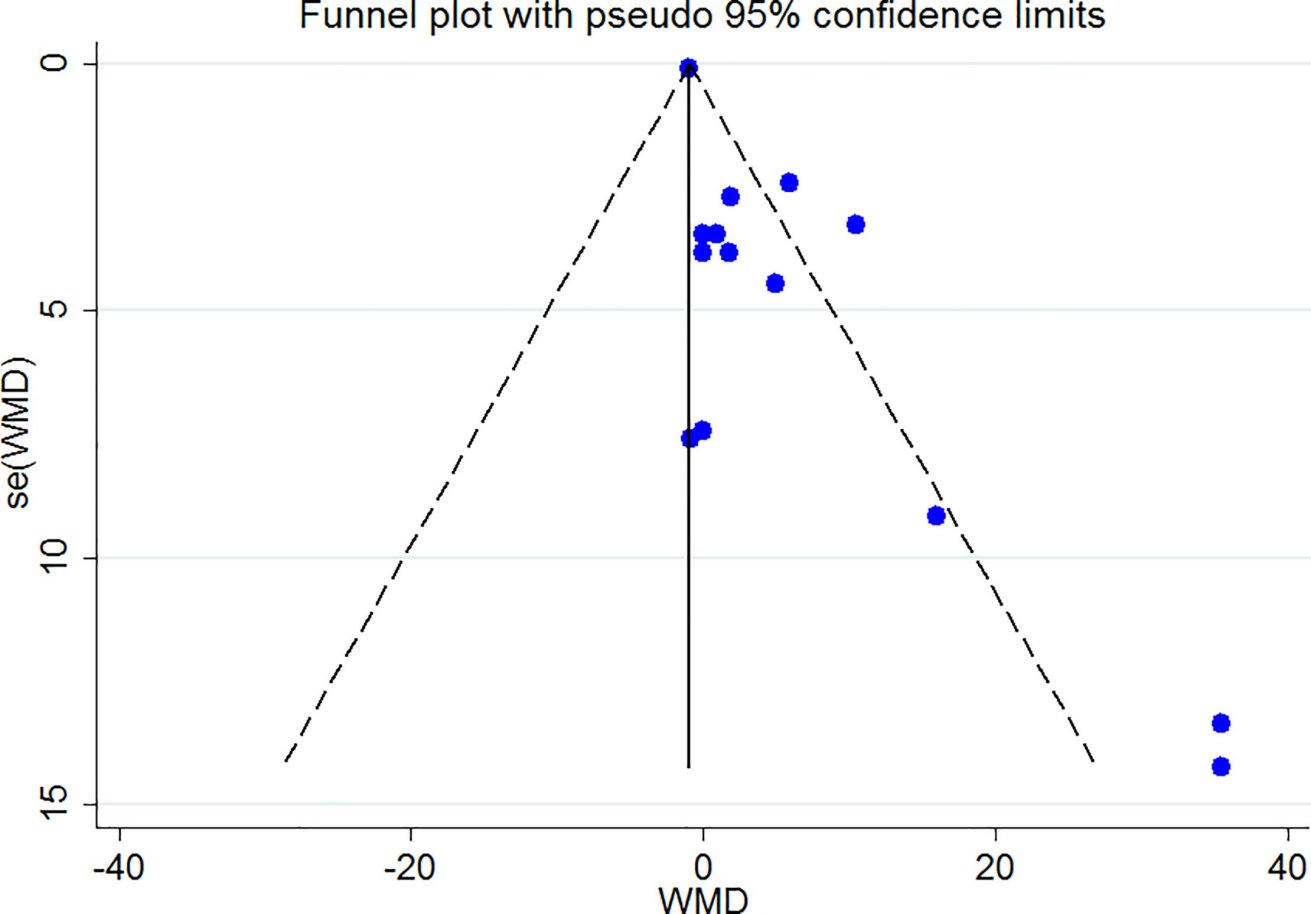
Begg's funnel plots (with pseudo 95% CIs) of the WMD versus the se (WMD) of the association between TG, PRAL and NEAP.

**Fig 14:**
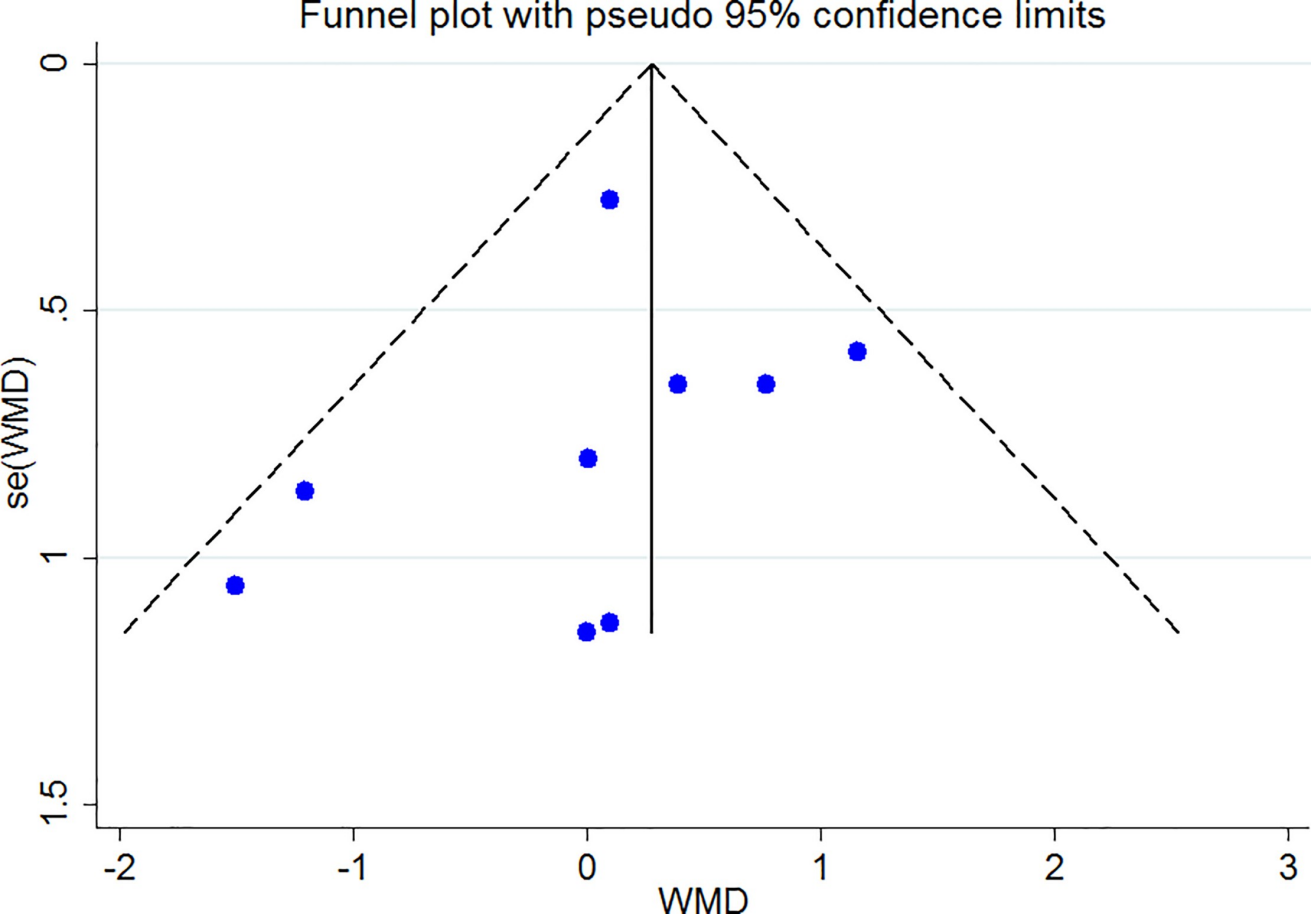
Begg's funnel plots (with pseudo 95% CIs) of the WMD versus the se (WMD) of the association between HDL, PRAL and NEAP.

**Fig 15:**
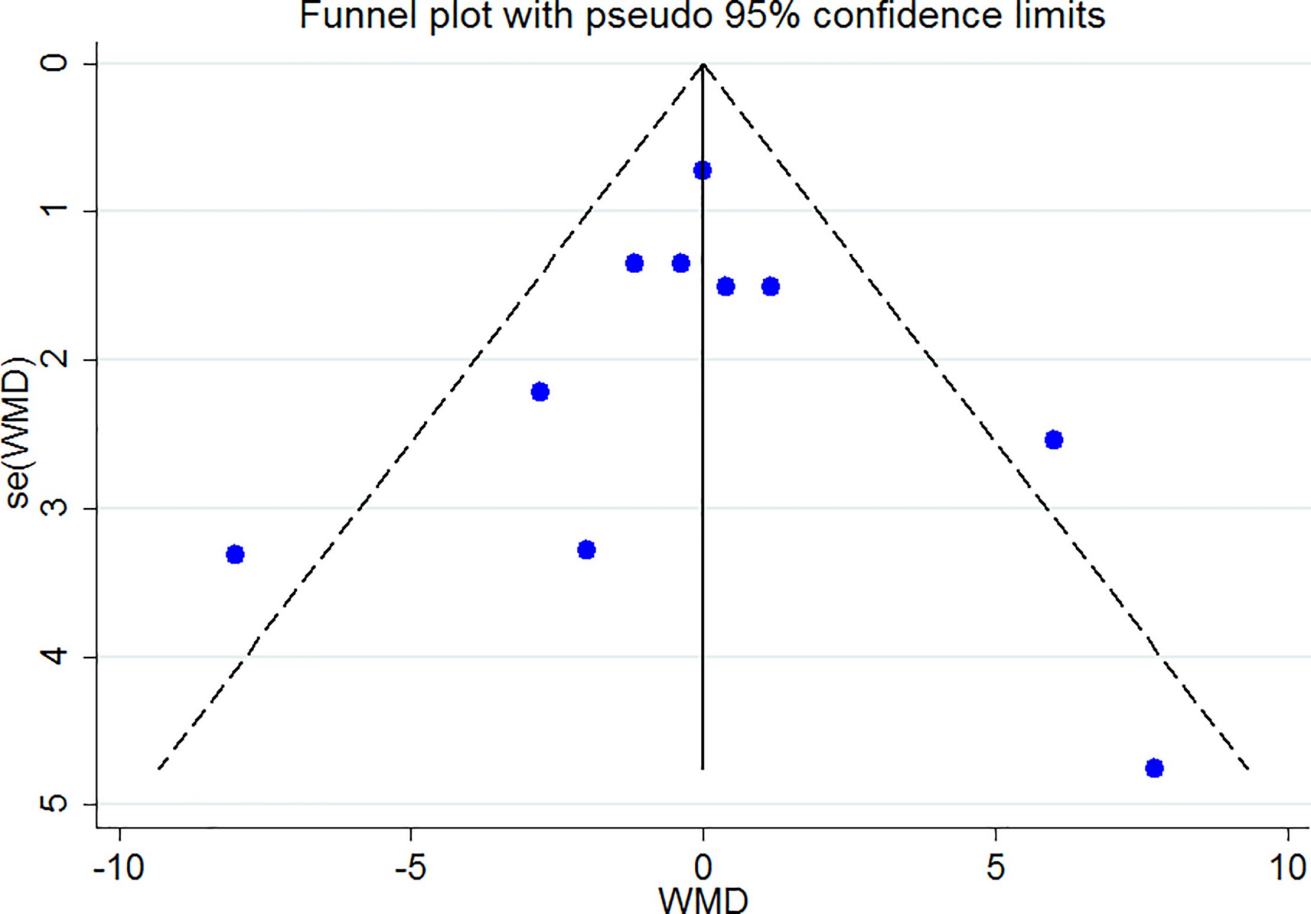
Begg's funnel plots (with pseudo 95% CIs) of the WMD versus the se (WMD) of the association between LDL, PRAL and NEAP.

The Funnel plots revealed moderate asymmetry (Figs 10–15). However, the Begg’s and Egger’s tests provided no evidence of substantial publication bias for all of the variables. The provided values are as follows: BMI, Egger’s test (P = 0.771) and Begg’s test (P = 0. 159); WC, Egger’s test (P = 0.246) and Begg’s test (P = 0. 615); TC, Egger’s test (P = 0.083) and Begg’s test (P = 0.10); TG, Egger’s test (P = 0.001) and Begg’s test (P = 0.701); HDL, Egger’s test (P = 0.649) and Begg’s test (P = 0.178); LDL, Egger’s test (P = 0.629) and Begg’s test (P = 0.531).

## Discussion

In the current meta-analysis, we summarized the results of studies reporting the association between PRAL, NEAP and body mass index, waist circumference, lipid profile and the prevalence of obesity. Accordingly, being in the highest category of PRAL scores was associated with higher TG and higher prevalence of obesity compared with lowest category. No association between BMI, WC and other serum lipids with PRAL or NEAP was observed. In subgroup analysis, increased PRAL scores in women and increased NEAP scores in men were associated with higher BMI. Animal foods including meat, fish, egg, chicken, cheese and also cereals are rich in sulfur containing amino acids, phosphorous and chloride are potentially acid formers; while vegetables and fruits high in malate, citrate and glutamate are potentially base formers therefore, animal based-foods and high contents in western diets are potentially considered as most important acid-producer diets and are associated with higher risk of insulin resistance, high blood pressure and diabetes as established in numerous works [10]. Accordingly, western dietary pattern with high dietary acid load content, is a potent inducer of central obesity and metabolic syndrome; several studies had revealed significant relationships between western dietary pattern and the increased risk of MetS, cholesterol and increased waist circumference and BMI. Accordingly, western dietary pattern with high content of red meat, eggs, and refined grains is associated with increased risk of obesity and increased levels of blood sugar, systolic blood pressure, triglycerides, and reduced levels of HDL [45–47]. Higher prevalence of obesity in higher categories of PRAL and NEAP could also be a attributed to the possible adipogenic effects of higher dietary acid load; in the study by Li et al. [48] among 29520 Chinese adults aged 18–70 years and higher prevalence of obesity in higher versus lower quintiles of PRAL was observed. Although we did not observed association between PRAL and NEAP with BMI in total analysis, however, in subgroup analysis of men and women separately, the PRAL-BMI association was significant for women and the NEAP-BMI association was significant for men. These gender-specific results might be due to the difference in the lean body mass; it has been demonstrate that higher dietary acid load reduces lean body mass only among women and not in men and finally leads to higher body fat synthesis [15] and that more alkalinogenic diets are associated with greater skeletal muscle mass among women [40]. Another possible explanation is the difference in sex-hormones affecting acid-base balance [49]. Acidosis leads to loss of muscle mass through reducing protein synthesis and increasing proteolysis and amino acid oxidation, mediated via the ubiquitin proteasome system or via in IGF-1 signaling alterations [50]. This impaired acid-base balance is possibly the reason of reduced calcaneal broadband ultrasound attenuation and bone density in women and not in men [39]. In other word, it will be better to evaluate the association between dietary acid load indices and fat mass or fat free mass as indicators of adiposity instead of BMI to better elucidate the obesity-dietary acid load associations. In the current meta-analysis we also observed a positive association between PRAL and TG. The underlying mechanisms of increased TG concentrations in higher scores of PRAL are not well elucidated; however, several proposed mechanisms might be raised cortisol secretion and reduced insulin sensitivity and secretion and their consequent lipid disorders [35]. As mentioned in the results section, dietary assessment tool and continent could be a source of heterogeneity among observed association. In the current meta-analysis, PRAL and NEAP calculation was based on self-reported data gathered by 24 hours recall method, 24 hours record method and food frequency questionnaire which may be a potential source of bias. Moreover, difference in the items of the FFQ might be a source of heterogeneity; as described previously, the FFQ items ranged from 63 to 168 items and the local foods in the FFQ could also affect the heterogeneity [51], although, almost all of the included studies used valid and reliable FFQs. FFQ covers a wide range of dietary ingredients and is more accurate than 24-hours recall method reflecting usual dietary intake in a short period of time; it has been confirmed that FFQ could be more helpful in evaluating the diet-disease relationships [52]. Another source of heterogeneity, the continent, presents the possible role of geographical distribution, genetic background and cultural factors influencing the association between dietary acid load and metabolic risk factors [53–57]. The current meta-analysis has several limitations and strengths; the current meta-analysis included the results of observational studies with the cross-sectional or cohort design which makes the causal inference impossible; although, the studies were large population-based studies with acceptable quality. Moreover, the PRAL and NEAP were calculated based on self-reported data gathered by 24 hours recall method, food record or food frequency questionnaire which might be potential sources of bias. However, our study, based on our knowledge, is the first meta-analysis evaluating the association between dietary acid load as both PRAL and NEAP scores with a wide range of obesity related parameters including BMI, WC, LDL, HDL, TG, TC and the prevalence of obesity. In conclusion, in the current meta-analysis, we found a positive association between TG and PRAL and a gender-specific associations between PRAL, NEAP and BMI while no association between dietary acid load and other parameters were reported.

## Supporting information

S1 File Literature review. (XLSX)

S2 File PRISMA 2009 checklist. (DOC)
